# Coordination assembly of 2D ordered organic metal chalcogenides with widely tunable electronic band gaps

**DOI:** 10.1038/s41467-019-14136-8

**Published:** 2020-01-14

**Authors:** Yanzhou Li, Xiaoming Jiang, Zhihua Fu, Qingqing Huang, Guan-E. Wang, Wei-Hua Deng, Chen Wang, Zhenzhu Li, Wanjian Yin, Banglin Chen, Gang Xu

**Affiliations:** 1 State Key Laboratory of Structural Chemistry, Fujian Institute of Research on the Structure of Matter, Chinese Academy of Sciences (CAS), 155 Yangqiao Road West, Fuzhou Fujian, 350002 China; 2 University of Chinese Academy of Sciences, No.19A Yuquan Road, Beijing, 100049 China; 3 College of Energy, Soochow Institute for Energy and Materials InnovationS (SIEMIS), Key Laboratory of Advanced Carbon Materials and Wearable Energy Technologies of Jiangsu Province, Soochow University, Suzhou, 215006 China; 4 Key Lab of Advanced Optical Manufacturing Technologies of Jiangsu Province & Key Lab of Modern Optical Technologies of Education Ministry of China, Soochow University, Suzhou, 215006 China; 5 Department of Chemistry, University of Texas at San Antonio, San Antonio, TX USA

**Keywords:** Coordination chemistry, Electronic properties and materials, Organic-inorganic nanostructures, Two-dimensional materials

## Abstract

Engineering the band gap chemically by organic molecules is a powerful tool with which to optimize the properties of inorganic 2D materials. The obtained materials are however still limited by inhomogeneous compositions and properties at nanoscale and small adjustable band gap ranges. To overcome these problems in the traditional exfoliation and then organic modification strategy, an organic modification and then exfoliation strategy was explored in this work for preparing 2D organic metal chalcogenides (OMCs). Unlike the reported organically modified 2D materials, the inorganic layers of OMCs are fully covered by long-range ordered organic functional groups. By changing the electron-donating ability of the organic functional groups and the electronegativity of the metals, the band gaps of OMCs were varied by 0.83 eV and their conductivities were modulated by 9 orders of magnitude, which are 2 and 10^7^ times higher than the highest values observed in the reported chemical methods, respectively.

## Abstract (web summary)

Here, the authors report a new class of 2D materials with inorganic metal chalcogenide layers covered by long-range ordered organic groups. The range of modulation on their band gaps and electrical conductivities are two times and 7 orders of magnitude better than the highest values reported so far.

## Introduction

**Fig. 1:**
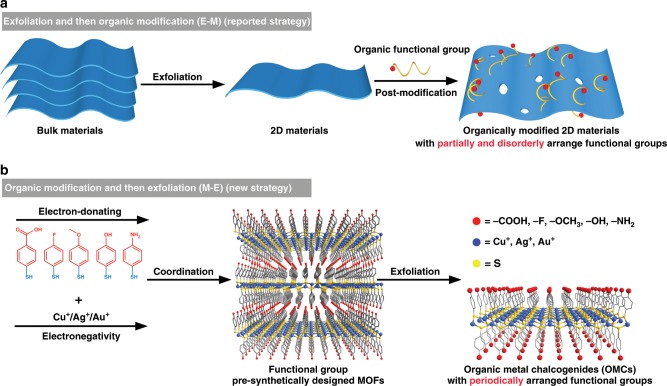
Comparison between new and reported synthetic strategies for 2D materials. **a** organic modification and then exfoliation (**M**–**E**) strategy for OMCs; **b** exfoliation and then organic modification (**E**–**M**) strategy for the known 2D materials.

Inorganic two-dimensional (2D) materials, such as graphene, graphene oxide (GO), transition metal dichalcogenides (TMDs), hexagonal boron nitride (h-BN) and black phosphorus (BP), have received unprecedented attention in recent years due to their unusual chemical and physical properties^1–8^. Electronic band gaps are determinative in the electronic transportation and optical properties of these 2D materials^9–11^. For example, graphene possesses a charge mobility 10 times higher than silicon and thus is desired to be used as a channel material in high-speed transistor. Its zero-band gap however limits its on-off ratio to <30^1,7^. Comparatively, a transistor made of a bi-layer graphene nanoribbon with a band gap of ~0.16 eV shows a dramatically boosted on-off ratio of 10^7^^11^. Besides the thickness control in this example, the band gap engineering of 2D materials has been widely explored by a number of chemical methods such as covalently bonding or physically adsorbing organic functional molecules, doping heteroatoms or alloying the second 2D materials into the primitive 2D semiconducting materials^10,12–31^. Among these methods, organic modification onto 2D materials by covalently bonding or physically adsorbing organic molecules not only possess the ability to modulate the band gap, but also to introduce functions related to organic molecule into 2D materials, such as better dispersion in solvent, extra light absorption and favorable affinity for target chemicals^4,15,32–34^. However, all organic modification methods reported so far belong to an exfoliation and then organic modification (**E-M**) strategy (Fig. 1a). The maximum modulated scope on the band gap by organic modification methods to date has been limited to 0.15 eV to date^15^. Secondarily, the organic groups on 2D materials are not homogeneously distributed, and this hinders the application of these materials. This work aims to develop a chemical method to address these two issues.

As has been demonstrated in the synthesis of metal-organic framework (MOF) and covalent-organic framework (COF) materials, the emerging reticular chemistry promises to develop multifunctional materials with predictable structures and thus tunable properties^35–39^. In fact, many functional MOF and COF materials have been produced for different applications ranging from gas separation, gas storage, heterogenous catalysis, photonics and drug delivery^40–43^. Such significant progress in material design and synthesis will certainly facilitate the discovery of other types of materials^44,45^.

In this work, we developed a metal-organic framework-directed organic modification and then exfoliation (**M-E**) strategy to prepare 2D organic metal chalcogenide (OMC) materials with a widely and finely adjustable band gap in two steps (Fig. 1b). First, a series of bulk iso-structural MOFs with layered structures were synthesized and structurally characterized; second, these MOF precursors were exfoliated to derive their related 2D materials or OMCs. OMCs have the general formula, **M(SPh-X)** (M = Cu^+^, Ag^+^, and Au^+^, respectively, HSPh is benzenethiol, X is the functional group *para* to the SH group and X = −COOH, –F, –OCH_3_, –OH, or –NH_2_). Distinct from the 2D materials modified by the earlier methodologies (Fig. 1b), OMCs prepared by this strategy are the first series of homogeneous inorganic 2D materials bearing periodically arranged organic functional groups. This structural feature facilitates wide adjustment in the band gaps and the electronic conductivity of OMCs by changing the electron-donating ability of organic functional groups and the electronegativity of metals.

## Results

### Synthesis and characterization

OMCs were all prepared with a similar method. Here, we describe the preparation of one single OMC, **Cu(SPh-OH)**, as an example. First, the parent bulk MOF of **Cu(SPh-OH)** was synthesized by the reaction between CuCl_2_·2H_2_O and 4-Hydroxybenzenethiol (**HSPh-OH**) in ethanol at 85 °C. Although, **Cu(SPh-OH)** has been synthesized as the crystallite powder^46^, a crystal that large enough for single crystal X-ray diffraction measurements was obtained by an *in-situ* redox reaction between the –SH on ligand and Cu^2+^. Mono- or few-layer **Cu(SPh-OH)** was prepared by a liquid exfoliation method. The optimum exfoliation conditions have been obtained by screening the type of solvent, the power and time for sonication, as well as the rate and period for centrifugation. Few-layer Cu(SPh-OH) can be obtained by sonicating the bulk in ethanol with the power of 135 W and 30 min and then centrifuging the suspension at 2000 rpm for 30 min. Other OMCs, **Cu(SPh-COOH)** (**HSPh-COOH** = 4-mercaptobenzoic acid), **Ag(SPh-COOH)**, **Au(SPh-COOH)**, **Ag(SPh-NH**_**2**_**)** (**HSPh-NH**_**2**_ **=** 4-aminobenzenethiol), **Ag(SPh-OH)**, **Ag(SPh-OCH**_**3**_**)** (**HSPh-OCH**_**3**_ **=** 4-methoxybenzenethiol), **Ag(SPh-F)** (**HSPh-F** = 4-fluorobenzenethiol), were obtained by the similar method but varying the metal sources and organic ligands (Fig. 1a). The structure of the parent bulk MOF of **Cu(SPh-OH)** was characterized by X-ray crystallographic analysis (Supplementary Table 1–3). Other OMCs are isostructural with **Cu(SPh-OH)**, and this was confirmed by their powder X-ray diffraction (PXRD) measurements (Supplementary Table 4). Although synthesized with organic ligands and metal ions by coordination, OMCs have unique 2D inorganic structures, which are different from the typical coordination polymers^45^. In **Cu(SPh-OH)**, each metal atom adopts a slightly distorted trigonal planar geometry coordinated with three μ_3_-bridging S atoms of the ligands. The Cu-S units form a honeycomb-like hexatomic ring [CuS] structures. Besides coordinating to metal atoms, each S atom covalently bonded to a benzene ring associated with one functional group (–NH_2_, –OH, –OCH_3_, –F, or –COOH) which extends out from the inorganic layer (Supplementary Fig. 1–9). In this work, both surfaces of OMCs are orderly and fully covered by the pre-designed functional groups, which have not been observed in the reported 2D materials and might be used to tune the electrical properties of OMCs homogeneously. Depending on the organic functional groups, the thickness of the mono-layer OMCs varies from 1.45 to 1.81 nm (Supplementary Fig. 2–9). The successful preparation of OMCs was further confirmed by FTIR, XPS and elemental analysis (Supplementary Fig. 11–18, Supplementary Fig. 19 and Supplementary Table 5). Thermogravimetric analyses demonstrated that these materials have high thermal stability up to 300 °C (Supplementary Fig. 20). Most of OMCs also showed good chemical stability in pH ranging from 3 to 11 for >12 h (Supplementary Fig. 21–28).

### Band gap modulation of OMCs

**Fig. 2:**
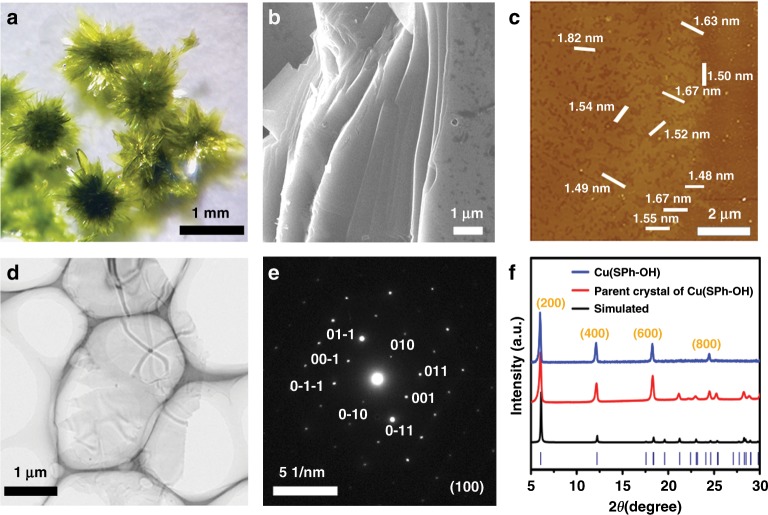
Synthesis and characterization of OMCs. **a** Optical photograph and **b** SEM of the parent bulk MOF of **Cu(SPh-OH)**; **c** AFM image of monolayer **Cu(SPh-OH)**; **d** TEM of **Cu(SPh-OH)** and **e**, its corresponding SAED pattern; **f** PXRD pattern of **Cu(SPh-OH)** and its parent bulk MOF crystal.

Figure 2a, b and Supplementary Fig. 29 show a typical optical photograph and SEM images of the parent bulk MOF of OMCs with laminar architectures. Atomic force microscopic (AFM) images clearly show that OMCs with smooth surfaces and homogeneous height from 1.48 to 4.30 nm were obtained, respectively (Fig. 2c and Supplementary Fig. 30). These thicknesses indicate 1–3 layers of OMCs. The transmission electron microscopy (TEM) images (Fig. 2d and Supplementary Fig. 31) further illustrates their nature as 2D materials. The crystal structures of OMCs were confirmed by selected area electron diffraction (SAED) and PXRD patterns (Fig. 2e and Supplementary Fig. 31). Typically, the SAED pattern in Fig. 2e shows bright and distinct spots which can be assigned very well to the indices of {0kl} face of **Cu(SPh-OH)**. The PXRD patterns (Fig. 2f and Supplementary Fig. 31) match well with the simulated patterns of OMCs. The results from AFM, TEM and PXRD measurements demonstrate that the prepared ultrathin nanosheets are highly crystalline OMCs.

**Fig. 3:**
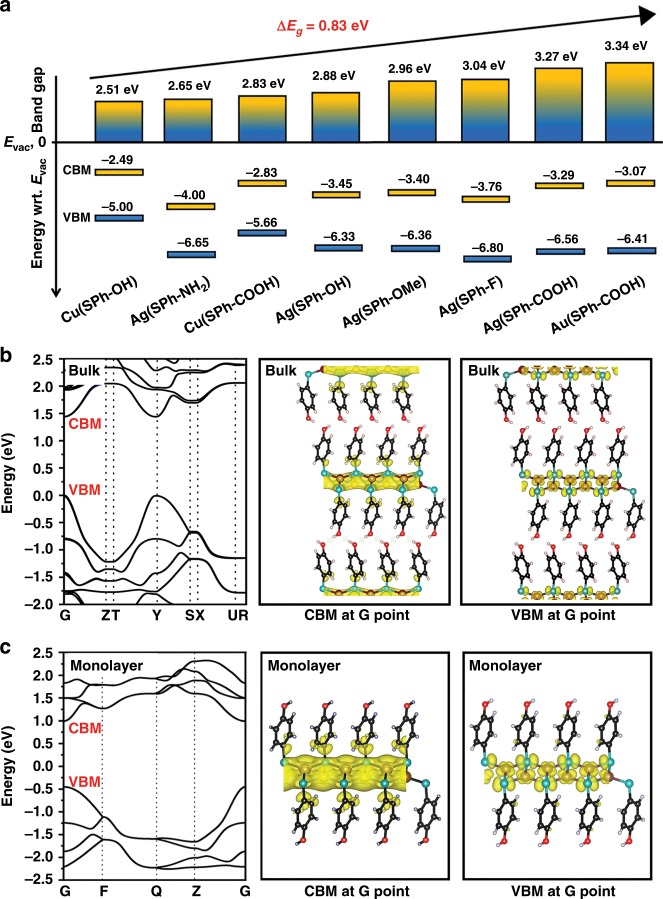
Band gap modulation and band structure calculation of OMCs. **a** The band gap of OMCs modulated by changing the components (calculated from UV–vis diffuse reflectance spectra); **b** calculated band structure of bulk **Cu(SPh-OH)** and its corresponding VBM and CBM at Gamma (G) point; **c** calculated band structure of monolayer **Cu(SPh-OH)** and its corresponding VBM and CBM at Gamma (G) point.

UV-Vis spectrophotometric measurements (Supplementary Fig. 32–33) reveal that OMCs possess high flexibility in modulation of their band gaps by changing their metal ions or organic functional groups: (1) with the same ligand, increasing the electronegativity of the metal ions results broaden band gap in the sequence of  the **Cu(SPh-OH)** (2.51 eV) < **Ag(SPh-OH)** (2.88 eV) and **Cu(SPh-COOH)** (2.83 eV) < **Ag(SPh-COOH)** (3.27 eV) < **Au(SPh-COOH)** (3.34 eV) (Fig. 3a and Supplementary Fig. 33a–b); (2) with the same metal ion, the band gap becomes narrower with the enhancement of electron-donating ability of organic functional groups on the benzene ring in the sequence of **Ag(SPh-NH**_**2**_**)** (2.65 eV) < **Ag(SPh-OH)** (2.88 eV) < **Ag(SPh-OCH**_**3**_**)** (2.96 eV) < **Ag(SPh-F)** (3.04 eV) < **Ag(SPh-COOH)** (3.27 eV) (Fig. 3a and Supplementary Fig. 33c). Before this work, alloying the second 2D materials into the primitive 2D materials reached the highest value (0.42 eV) of band gap modulation in all reported chemical methods^22^. The band gaps of OMCs were adjusted by 0.83 eV, which is the highest value reported to date: 5.5 times higher than the highest value (0.15 eV) realized by **E-M** strategy^22^; twice as high as the highest value achieved by all chemical methods^22^.

Ultraviolet photoelectron spectroscopy (UPS) measurements (Fig. 3a and Supplementary Fig. 34) show that the Fermi levels of all OMCs deviate from the middle of the band gap and are close to the valence band (VB), which suggests OMCs are p-type 2D materials^47,48^. Density functional theory (DFT) calculations were performed to study the thickness-dependent band structure of OMCs. As shown in Fig. 3b–c, Supplementary Fig. 35–48 and Supplementary Table 6, OMCs are featured with nearly unchanged band gaps when decreasing layer number from bulk to single layer. On the other hand, the valence band maximum (VBM) and conduction band minimum (CBM) at Gamma (G) point of OMCs show no shift when decreasing the number of layers. These features make their properties independent of the number of layers and facilitate the characterizations and applications of OMCs.

### Conductivity modulation of OMCs

**Fig. 4:**
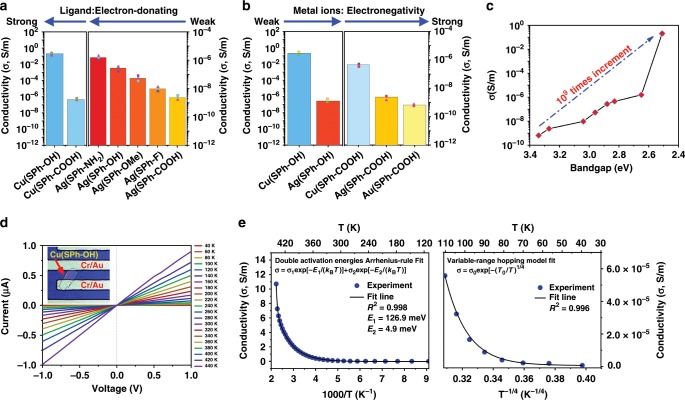
Conductivity studies of OMCs. **a, b** Electronic conductivity of OMCs arranged according to the electron-donating ability of organic ligands and electronegativity of metal ions, respectively; **c** Band gap dependent conductivity of OMCs (Error bar, mean±s.d.); **d** Optical photograph of 25 nm thick **Cu(SPh-OH)** inbetween Cr/Au electrodes and its temperature-dependent *I*–*V* curves; **e** Variable temperature conductive measurements of **Cu(SPh-OH)** in **d**; σ-T^−1^ plot between 110 and 440 K (left); the solid line is the fitting curve based on double activation energies equation; σ-T^−1/4^ between 40 and 100 K (right); the solid line is the fitting curve based on Mott variable-range hopping model.

Conductivity measurements were performed with pressed pellets of OMCs by the standard two-contact probe method^49^. Average room temperature conductivity values from 3 samples for each OMCs were obtained and plotted in Fig. 4a, b, Supplementary Fig. 49 and Supplementary Table 7. **Cu(SPh-OH)** showed the highest conductivity of 0.2 S m^−1^ in OMCs. It was found that the conductivity of OMCs increases with a decrease of their optical band gaps and can be tuned over 9 orders of magnitude (Fig. 4c). This value is 7 order of magnidude higher than the highest value modulated by the reported chemical methods^50,51^. Chemically, the conductivity of OMCs can be modulated by changing the inorganic or organic components: (1) the conductivity increases by enhancing the electron-donating ability of organic functional group in the sequence **Ag(SPh-NH**_**2**_**)** > **Ag(SPh-OH)** > **Ag(SPh-OCH**_**3**_**)** > **Ag(SPh-F)** > **Ag(SPh-COOH)** (Fig. 4a); (2) the conductivity increases by weakening the electronegativity of metal ions in the sequence **Cu(SPh-OH)** > **Ag(SPh-OH)** and **Cu(SPh-COOH)** > **Ag(SPh-COOH)** > **Au(SPh-COOH)** (Fig. 4b).

## Discussion

The conductivity of OMCs modulated by organic functional groups might be explained by a chemical gating effect, which is similar to the electrical gating in FETs (Supplementary Fig. 50)^52–54^. As shown in Supplementary Fig. 50a, when applying a negative gating voltage (V_G_) to a *p*-type FET, an electric dipole with a direction pointing from gate electrode to semiconductor in the dielectric layer is created. Attracted by this electric dipole, additional charge carriers, or holes, are injected from the source electrode into the semiconductor, increasing its conductivity. As shown in Supplementary Fig. 50b, the inorganic layers in OMCs are *p*-type semiconductors with a hole as their dominant charge carrier. The electron-donating functional groups on the benzene ring can be considered as a gating voltage (V_G_′) and create an electric dipole with the direction from electron-donating functional groups pointing to S atoms. Similar to the case in FETs, V_G_′ would induce additional holes from electrodes to inject into OMCs due to the chemically created electric dipole. The stronger electron-donating ability results in a stronger electric dipole and thus the more injected holes and the higher conductivity of OMCs. On the other hand, a metal ion possessing smaller electronegativity has weaker binding force to outer electrons and its outer electrons could be more easily delocalized to obtain higher conductivity^55^. Coinage metal ions have the electronegativity values in the sequence of Cu^+^ (1.90) < Ag^+^ (1.93) < Au^+^ (2.54), which explains the fact that the conductivity of OMCs with the same ligand is in the sequence of Cu–OMCs > Ag–OMCs > Au–OMCs.

**Cu(SPh-OH)** with 25 nm in thickness was selected to study the electrical properties in more detail. (Fig. 4d) Its conductivity was measured in a direction parallel to the inorganic layer (h00), and showed a value of 1.2 S m^−1^, which is 6 times higher than that of the pressed pellet (0.2 S m^−1^) and is due to the lack of a grain boundary (Fig. 4d). The conduction mechanisms of **Cu(SPh-OH)** were studied by variable-temperature conductivity measurements. Figure 4e shows the measured conductivity plotted as a function of reciprocal temperature for **Cu(SPh-OH)** between 110 K and 440 K. The conductivity increases when raising the temperature, reaching 10.71 S m^−1^ at 440 K. It clearly manifests that a double activation energy equation can fit our experimental data well with R^2^ as high as 0.998 in this temperature range. This result indicates a typical Arrhenius-type behavior and can be explained by the charge transport arising from the thermal activation conduction processes^56^. This observation indicates that there are a group of intermediately deep acceptors with an activation energy of E_1_ ≈ 126.9 meV and a group of shallow acceptors with an activation energy of E_2_ ≈ 4.9 meV. At the relative higher temperatures, both the shallow and the intermediately deep acceptors are consecutively excited to the valence band, which represents a band conduction process^56^. As the temperature decrease to ~110 K, the charge carriers are gradually frozen out and the acceptors may no longer be effectively excited to the VB, resulting in another conduction transport mechanisms at temperature below 110 K. Figure 4e shows that the temperature dependent conductivity of **Cu(SPh-OH)** follows the Mott’s law in the range 110–40 K, indicating a Mott variable-range-hopping conduction in the states with energy concentrated near the Fermi level^57,58^. Such a conduction mechanism demonsration can also be found in the other coordination polymer and p-type semiconductor^59,60^.

In conclusion, a MOF-directed **M-E** strategy has been explored to prepare OMCs, a type of 2D semiconductive materials. Compared with the organically modified 2D material prepared by the traditional **E-M** strategy, OMCs possess much wider adjustable band gap range and homogeneous compositions and properties at nanoscale. These advantages are attributed to the unique structure of OMCs, in which an inorganic metal chalcogenides layer is fully covered by periodically arranged organic functional groups. This structural feature makes OMCs the first series of homogeneous 2D materials with organically modified surfaces. By varying the electron-donating ability of the organic functional groups and the electronegativity of the metals, the band gap of OMCs was modulated by 0.83 eV. By decreasing the band gap, the electrical conductivity of OMCs increased over 9 orders of magnitude. The band gap and conductivity modulations are 2 and 10^7^ times higher than the highest values observed in the reported chemical methods, respectively. This MOF-directed preparation strategy shows great potential in the design of 2D materials with widely tunable band gaps. Moreover, the variable and long-range ordered organic functional groups on the surface make this type of 2D materials very appealing in the applications in chemistry, physics and biology.

## Methods

### Materials

All reagents were purchased commercially and used without further purification. The ligands: 4-Mercaptophenol (97%), 4-Methoxythiophenol (98%) and 4-Aminothiophenol (≥98%) were purchased from Aladdin. Whereas, the ligand 4-Fluorothiophenol (98%) was purchased from Macklin. 4-Mercaptobenzoic acid (90%) was purchased from Adamas Reagent, Ltd. China. Metal salts and metallic oxides: Silver nitrate (AR, ≥99.8%), Sodium hydroxide (AR, ≥96.0%), Tetrachloroauric (III) acid hydrate (AR, ≥47.8%), Copper (II) chloride dehydrate (AR, ≥99.0%) were purchased from Sinopharm Chemical Reagent Co., Ltd, China; Copper (I) oxide (AR, 97.0%) was purchased from Aladdin. Solvents and liquid reagents: Acetonitrile (AR, ≥99.0%), and Ethanol (AR, ≥99.7%) were purchased from Sinopharm Chemical Reagent Co., Ltd, China. Water was purified using the Milli-Q purification system. Hydrogen chloride (AR, 36.0–38.0%, *w*%) were also purchased from Sinopharm Chemical Reagent Co., Ltd, China.

### Synthetic procedure of single crystal of Cu(SPh-OH)

4-hydroxythiophenol (0.0836 g, 0.663 mmol) and CuCl_2_·2H_2_O (0.0158 g, 0.093 mmol) were dissolved in EtOH (5 mL) at room temperature. The mixture was sonicated for 1 h at room temperature, then 1.25 M NaOH was drop added. The solution was then heated to 85 °C for 2 weeks and gave light yellow sheet-like rectangle shape crystals. The yellow crystals were washed with ethanol, water, ethanol and finally diethyl ether.

### Synthesis of the parent microcrystal of Cu(SPh-OH)

4-hydroxythiophenol (0.0850 g, 0.674 mmol) and CuCl_2_·2H_2_O (0.0243 g, 0.143 mmol) were dissolved in EtOH (5 mL) at room temperature. The mixture was sonicated for 1 h at room temperature, then 1.25 M NaOH was drop added. The solution was then heated to 85 °C for 12 days and gave yellow sheet-like nanosheets. The yellow nanosheets were washed with ethanol, water, ethanol and finally diethyl ether. Then the Cu(SPh-OH) nanosheets were collected by suction filtration.

### Synthesis of the parent microcrystal of Cu(SPh-COOH)

Cu_2_O (0.0210 g, 0.147 mmol) and 4-Mercaptobenzoic acid (0.0677 g, 0.439 mmol) were mixed in water (5 mL) at room temperature, then 12 M HCl was drop added into the turbidity solution. Then the mixture was sonicated for 30 min. The solution was then heated to 85 °C for 10 days in the oven and gave pale yellow sheet-like nanosheets. The pale yellow nanosheets were washed with ethanol, water, ethanol and finally diethyl ether. Then the Cu(SPh-COOH) nanosheets were collected by suction filtration and dried under vacuum at 70 °C overnight.

### Synthesis of the parent microcrystal of Ag(SPh-OH)

4-hydroxythiophenol (0.1095 g, 0.868 mmol), AgNO_3_ (0.0210 mg, 0.124 mmol) were dissolved in water (5 mL) at room temperature, then cautiously add, dropwise triethylamine. Then the mixture was sonicated for 30 min. Later, the reactor was heated at 85 °C for 7 days and then allowed to cool to room temperature. The nanosheets were obtained, washed with ethanol, water, ethanol and finally diethyl ether then dried under vacuum at 70 °C overnight.

### Synthesis of the parent microcrystal of Ag(SPh-NH_2_)

4-Aminothiophenol (0.1752 g, 1.399 mmol) in a 50 mL beaker was dissolved in 25 mL water; the mixture was stirring 10 min, then add the solution of AgNO_3_ (0.0724 g, 0.426 mmol) in 5 mL water to it. The color of the mixture changes from croci to light yellow. Later, the mixture was heated in water bath to 100 °C under stirring, and then cautiously add triethylamine in it. After boiling 2 h, the solution was allowed to cool to room temperature. A large amount of high quality, crystalline, dark yellow nanosheets of Ag(SPh-NH_2_) were obtained, and washed with ethanol, water, ethanol and finally diethyl ether, and then dried under vacuum at 70 °C overnight.

### Synthesis of the parent microcrystal of Ag(SPh-OMe)

4-Methoxythiophenol (0.1104 g, 0.787 mmol) and 0.5 mL triethylamine were dissolved in 3 mL acetonitrile, and AgNO_3_ (0.0454 g, 0.267 mmol) was dissolved in 2 ml acetonitrile. Then 2 mL AgNO_3_ solution was added to the ligand solution. The mixture solution was sealed in a 7 mL vital container, which was heated at 85 °C for 2 days and then cooled down to room temperature. The nanosheets of Ag(SPh-OMe) were collected and washed with ethanol, water, ethanol and finally diethyl ether, and dried under vacuum at 70 °C overnight to give the product.

### Synthesis of the parent microcrystal of Ag(SPh-F)

4-Fluorothiophenol (0.1220 g, 0.952 mmol) and 1 mL triethylamine were dissolved in 10 mL acetonitrile in a 50 mL beaker; AgNO_3_ (0.0514 g, 0.303 mmol) was dissolved in 5 ml acetonitrile, then was cautiously added to the ligand solution. Then, the mixture was heated at 95 °C for 1 h, straight after cooling the product in an ice bath. A large amount of high quality, crystalline, silver nanosheets of Ag(SPh-F) was obtained, and washed with ethanol, water, ethanol and finally diethyl ether then dried under vacuum at 70 °C overnight.

### Synthesis of the parent microcrystal of Ag(SPh-COOH)

4-Mercaptobenzoic acid (0.1000 g, 0.649 mmol) was dissolved in 10 mL water in a 50 mL beaker, then drop 1 M KOH into the solution; AgNO_3_ (0.0529 g, 0.311 mmol) was dissolved in 5 ml water, then was cautiously added to the ligand solution. Then the solution was heated at 95 °C for 1 h, straight after cooling the product in an ice bath. A large amount of high quality, crystalline, white nanosheets of Ag(SPh-COOH) was obtained, and washed with ethanol, water, ethanol and finally diethyl ether and then dried under vacuum at 70 °C overnight.

### Synthesis of the parent microcrystal of Au(SPh-COOH)

First, 4-mercaptobenzoic acid (0.148 g, 0.960 mmol) was dissolved in a small amount of ethanol. Then NaOH (0.040 g, 1.0 mmol) was added to the 4-mercaptobenzoic acid aqueous solution. The obtained mixtures were transferred to 20 mL volumetric flasks and brought to volume by ethanol. Hence, 0.05 M sodium 4-mercaptobenzoic acid solution was obtained. Second, HAuCl_4_ aqueous solution (10 mL, 25.3 mM) was added to 60 mL ethanol in an 100 mL beaker and heated to boil on a hot plate. Then sodium 4-mercaptobenzoic acid solution (20 mL, 0.05 M) was injected, which generated white precipitates immediately. And the color of the solution changes from yellow to colorless. Boil the mixture for 2 h, then products were purified by 12,000 rpm centrifugation to remove the residues before characterization, washed with absolute ethanol for two times and then distilled water for two times. Finally, the products were dried under vacuum at 70 °C overnight.

### OMCs preparation

The post-optimized exfoliation procedures of the OMCs are listed as follows:

1. Cu(SPh-OH) was added into 5 mL EtOH in an 8 mL vial with an initial concentration of 0.5 mg/mL in a sonic bath at power output of 135 W for 30 min. Centrifugation at 2000 rpm for 30 min. The top of 2/3 was collected by pipette.
2. Cu(SPh-COOH) (1 mg/mL) in 10 mL vial was sonicated with 200 W output power for 60 min in water. Then centrifugation at 5000 rpm for 30 min. The top of 2/3 was collected by pipette.
3. Ag(SPh-OH) was added into 10 mL EtOH in a 20 mL vial with an initial concentration of 3 mg/mL in a sonic bath at power output of 200 W for 60 min. Centrifugation at 2000 rpm for 30 min. The top of 2/3 was collected by pipette.
4. Ag(SPh-NH_2_) was added into 50 mL EtOH in a 100 mL conical flask with an initial concentration of 1 mg/mL in a sonic bath at power output of 200 W for 45 min. Centrifugation at 5000 rpm for 30 min. The top of 2/3 was collected by pipette.
5. Ag(SPh-OMe) was put into EtOH in a 7 mL vial with concentration 1 mg/mL. Then sonicated it at 150 W for 120 min, finally centrifugation for 30 min at 500 rpm. The top of 2/3 was collected by pipette.
6. Ag(SPh-F) was added to EtOH (5 mL in a 7 mL vial) with an initial concentration of 1 mg/mL. It was sonicated using a sonic bath for 30 min, with a nominal power output of 200 W. After sonication, the dispersions were allowed to centrifuge them at 2000 rpm for 30 min. The top of 2/3 was collected by pipette.
7. Ag(SPh-COOH) was added to EtOH (10 mL in a 20 mL vial), with an initial concentration of 1 mg/mL and bath sonicated with a nominal power output of 200 W for 45 min. Centrifugation at 12000 rpm for 30 min. The top of 2/3 was collected by pipette.
8. Au(SPh-COOH) was added into 20 mL cylindrical vial with the concentration of 1 mg/mL in water and a power sonic bath for 45 min. The resulted dispersions were centrifuged at 12000 rpm for 30 min.

### Characterization

The FT-IR spectra were recorded on a Bruker VERTEX70 FT-IR spectrometer in 4000–400 cm^−1^ region using KBr pellets. PXRD patterns were recorded on a Rigaku MiniFlex II diffractometer and a Rigaku Miniflex 600 diffractometer using Cu Kα radiation by keeping the powdered sample on a silicon substrate, from 5 to 65°. Thermogravimetry analysis was made on a Netzsch STA449C simultaneous TGDTA/DSC apparatus under air atmosphere with each sample heated in an Al_2_O_3_ crucible at a heating rate of 10 K min^−1^. The elemental analyses of C, H, and N were measured on an Elementar Vario EL III microanalyzer. UV–vis absorption/reflection spectroscopy was performed with a Lambda 950 using BaSO_4_ background in a quartz plate. The morphology and microstructure of the fabricated materials were characterized by a field emission scanning electron microscopy, JEOL model JSM-6700 FE-SEM, operating at an accelerating voltage of 1.5 or 5.0 kV. TEM images were obtained on a JEOL-2010 transmission electron microscope at an acceleration voltage of 200 kV. AFM measurements were performed using Bruker dimension ICON scanning probe microscope. The conductivity of the powder pellets was measured with a two-probe method using Keithley 4200 semiconductor analyzer. The pellets were pressed at a pressure of ≈1 GPa. The pellets were connected to the system by gold wires with both of their surfaces covered by silver paste. The variable temperature electrical measurements were performed in vacuum at different temperatures using a Lakeshore probe station. The surface chemical analysis was investigated by X-ray photoelectron spectroscopy (XPS) on a Thermo Scientific ESCALAB 250 Xi XPS system. Two different types of low power sonic baths were used; KQ5200DE (200 W, 40 kHz), KQ3200DE (150 W, 40 kHz) from Kunshan Ultrasonic Instrument. Co., LTD, China.

### X-ray data collection and crystal structure determination

The intensity data sets were collected for parent single crystal of Cu(SPh-OH) on a Rigaku Saturn 70 CCD diffractometer equipped with graphite-monochromated Mo Kα radiation (λ = 0.71073 Å) at 293 K. The data sets were reduced with the Crystal Clear program. An empirical absorption correction was applied using the multi-scan method. The structures were solved by direct methods using the Siemens SHELXL package of crystallographic software^61^. The difference Fourier maps were created on the basis of these atomic positions to yield the other non-hydrogen atoms. The structures were refined using a full-matrix least-squares refinement on *F*^2^. All non-hydrogen atoms were refined anisotropically. The hydrogen atoms were included in their calculated positions and refined riding on the respective parent atoms.

### Structural simulation using powder XRD data

(1) Sample Preparation and Data Collection: microcrystals Cu(SPh-COOH), Ag(SPh-NH_2_), Ag(SPh-OH), Ag(SPh-OMe), Ag(SPh-F), Ag(SPh-COOH), and Au(SPh-COOH) were freshly prepared and were mechanically ground into fine powders. The powder was side-loaded onto a glass holder to form a flat surface (ca. 14 mm (diameter) ×0.5 mm (depth)). Replicate X-ray diffraction datasets were collected on a Rigaku MiniFlex II diffractometer. X-ray diffractometer (λ(CuKα) = 1.5418 Å). Data collection was performed with the following: 2*θ* range = 5–50°, step size = 0.02° in 2*θ*, speed of scan = 10 s/step. (2) Structure Simulation: The crystal structures of Cu(SPh-COOH), Ag(SPh-NH_2_), Ag(SPh-OH), Ag(SPh-OMe), Ag(SPh-F), Ag(SPh-COOH), and Au(SPh-COOH) were firstly created based on the structure model of Cu(SPh-OH), and then the cell parameters were refined against PXRD patterns via Le Bail refinements using the Jana package^62^. Finally, the geometry optimization calculations based on density functional theory (DFT) were performed to get precise positions of all atoms in their structures using the CASTEP module implemented in the Materials Studio package^63,64^.

### DFT calculation of Cu(SPh-OH)

The structure of Cu(SPh-OH) was optimized by using the density-functional theory (DFT) method implemented in the Vienna ab initio simulation package (VASP). Both the parent crystal and mono-layer of Cu(SPh-OH) were calculated. The projected augmented wave (PAW) pseudopotentials and the general gradient approximation (GGA) parametrized by Perdew–Burke–Ernzerhof (PBE) was used as the exchange correlation functional. The wave functions at each k-point were expanded with a plane wave basis set and the kinetic cutoff energy is 520 eV. The electron occupancies were determined according to a Methfessel-Paxton scheme with an energy smearing of 0.2 eV. All structures were optimized by a conjugate gradient method until the force component on every atom was <0.01 eV/Å.

### DFT calculations of other OMCs

The structure of OMCs was optimized by using the density-functional theory (DFT) method implemented in the Vienna *ab initio* simulation package (VASP). The bandgaps of OMCs were calculated. The projected augmented wave (PAW) pseudopotentials and the general gradient approximation (GGA) parametrized by Perdew–Burke–Ernzerhof (PBE) was used as the exchange correlation functional. The wave functions at each k-point were expanded with a plane wave basis set and the kinetic cutoff energy is 460 eV. All structures were optimized by a conjugate gradient method until the force component on every atom was <0.01 eV/Å.

### Electronic properties of OMCs

Electrical conductivity, σ, is obtained by fitting the linear region of the current–voltage curves to Ohm’s law. For the bulk measurements, a 2-contanct probe method was used. Firstly, 5 mg of each powdered samples were pressed into cylindrical pellets with diameter of 2.5 mm and thickness ~0.5 mm, respectively. Then, the both round surfaces of the pellets were covered by silver paste and connected to the semiconductor analysis system (4200SCS, Keithley) by gold wires (diameter: 50 µm). The electrical conductivity of the samples was calculated based on Ohm’s law. For the thin flake measurement, a miro-electrode based 2-contanct probe method was used. Firstly, the as-exfoliated Cu(SPh-OH) nanosheets were dispersed into EtOH and then dropped onto a Si wafer (~500 µm in thickness) with a 300 nm SiO_2_ layer. Patterned electrodes were prepared with a standard electron beam lithography process (ELPHY Plus, Raith GmbH) and 10 nm Cr/50 nm Au were deposited on the top of the thin flake by a thermal evaporation (Nexdap, Angstrom Engineering). Cr layer was deposited for adhesion and Au layer was used as electrode. The contact areas at both end of the thin flake are ~2 and 20 μm^2^, respectively. The electrical characterization was performed by a cryogenic probe station (CRX-6.5 K, Lakeshore) connected to semiconductor analysis system (4200SCS, Keithley) at various temperature.

## Supplementary information

Supplementary Information

## Data Availability

The authors declare that all data supporting the findings of this study are available within the paper and its supplementary information files. The X-ray crystallographic coordinates for structure reported in this Article has been deposited at the Cambridge Crystallographic Data Centre (CCDC), under deposition number CCDC 1900345. Any further relevant data are available from the authors upon reasonable request.
