# Supplementary material for: Coordination assembly of 2D ordered organic metal chalcogenides with widely tunable electronic band gaps
Source: Nat Commun. 2020 Jan 14;11:261. doi: 10.1038/s41467-019-14136-8 (PMC6959344; doi:10.1038/s41467-019-14136-8)
Supplement: Supplementary file 1 — Supplementary Information [file 41467_2019_14136_MOESM1_ESM.pdf]

Supporting information

Yanzhou Li et al. Coordination Assembly of 2D Ordered Organic Metal Chalcogenides with Widely Tunable Electronic Band Gaps

Supplementary Table 1. Crystal data and structure refinement.

**Compound**	**Cu(SPh-OH)**
State	Single crystal
CCDC number	1900345
Empirical formula	$C_{13.16}H_{8.88}Cu_2O_{2.2}S_2$
M_r	393.42
Crystal system	Orthorhombic
Space group	$Pmn2_1$
a (Å)	3.995(2)
b (Å)	14.463(8)
c (Å)	5.119(3)
α (°)	90
β (°)	90
γ (°)	90
V ($Å^3$)	295.8(3)
Z	1
ρ_{calc} (g/cm^3)	2.209
μ /mm^{-1}	3.939
F(000)	195.4
λ (Å)	0.71073
2Θ range for data collection/°	8.446 to 54.872
Number of reflections	1255
Index ranges	$-5 \le h \le 3$,
	$-17 \le k \le 13$,
	$-6 \le l \le 6$
Independent reflections	640 [R_{int} = 0.0570, R_{sigma} = 0.0700]
Data/restraints/parameters	640/1/77
Goodness-of-fit on F^2	0.972
Final R indexes [I>=2σ (I)]	R_1 = 0.0442, wR_2 = 0.0999
Final R indexes [all data]	R_1 = 0.0546, wR_2 = 0.1038

Supplementary Table 2. Bond lengths for the parent single crystal of Cu(SPh-OH).

Atom	Atom	Length (Å)	Atom	Atom	Length (Å)
Cu1	S3[1]	2.2541(16)	C6	C8	1.3900
Cu1	S3[2]	2.2541(16)	C6	C5	1.3900
Cu1	S3	2.276(3)	C8	C3	1.3900
S3	C2	1.786(7)	C3	C2	1.3900
S3	Cu1[3]	2.2541(16)	C2	C7	1.3900
S3	Cu1[4]	2.2541(16)	C7	C5	1.3900
O2	C6	1.357(12)			

[1] 1/2-X, 2-Y, 1/2+Z; [2] -1/2-X, 2-Y, 1/2+Z; [3] -1/2-X, 2-Y, -1/2+Z; [4] 1/2-X, 2-Y, -1/2+Z

Supplementary Table 3. Bond angles for the parent single crystal of Cu(SPh-OH).

Atom-Atom-Atom	Angles (°)	Atom-Atom-Atom	Angles (°)
S3[1]-Cu1-S3[2]	124.79(13)	O2-C6-C5	117.9(8)
S3[1]-Cu1-S3	117.60(7)	C8-C6-C5	120.0
S3[2]-Cu1-S3	117.60(7)	C3-C8-C6	120.0
C2-S3-Cu1[3]	108.2(5)	C8-C3-C2	120.0
C2-S3-Cu1[4]	117.7(4)	C3-C2-C7	120.0
Cu1[3]-S3-Cu1[4]	124.79(13)	C3-C2-S3	123.7(5)
C2-S3-Cu1	111.9(3)	C7-C2-S3	116.3(5)
Cu1[3]-S3-Cu1	94.87(8)	C5-C7-C2	120.0
Cu1[4]-S3-Cu1	94.87(8)	C7-C5-C6	120.0
O2-C6-C8	122.0(8)		

[1] 1/2-X,2-Y,1/2+Z; [2] -1/2-X,2-Y,1/2+Z; [3] -1/2-X,2-Y,-1/2+Z; [4] 1/2-X,2-Y,-1/2+Z

Supplementary Table 4. Brief crystal data parameters.

**Compound**	**Cu(SPh-OH)**	**Cu(SPh-COOH)**	**Ag(SPh-NH$_2$)**	**Ag(SPh-OH)**
State	Powder	Powder	Powder	Powder
Empirical formula	C_6H_5CuOS	$C_7H_5CuO_2S$	C_6H_6AgNS	C_6H_5AgOS
M_r	188.71	216.73	232.05	233.04
Crystal system	Orthorhombic	Orthorhombic	Orthorhombic	Orthorhombic
Space group	$P2_12_12_1$	$P2_12_12_1$	$P2_12_12_1$	$P2_12_12_1$
a (Å)	28.915(1)	34.564	34.053	30.278
b (Å)	5.1255(3)	5.572	5.150	5.122
c (Å)	4.0006(2)	4.041	4.053	3.983
α (°)	90	90	90	90
β (°)	90	90	90	90
γ (°)	90	90	90	90
V (Å^3)	592.9(1)	778.26	710.79	617.75
R_p, R_{wp},	3.80, 5.57	6.38, 10.21	10.37, 14.00	9.37, 13.83
GOOF	1.22	2.19	1.49	1.63

Supplementary Table 4. Brief crystal data parameters. (continued table)

**Compound**	**Ag(SPh-OMe)**	**Ag(SPh-F)**	**Ag(SPh-COOH)**	**Au(SPh-COOH)**
State	Powder	Powder	Powder	Powder
Empirical formula	C_7H_7AgOS	C_6H_4AgFS	$C_7H_5AgO_2S$	$C_7H_5AuO_2S$
M_r	247.06	235.03	261.05	350.15
Crystal system	Orthorhombic	Orthorhombic	Orthorhombic	Orthorhombic
Space group	$P2_12_12_1$	$P2_12_12_1$	$P2_12_12_1$	$P2_12_12_1$
a (Å)	34.352	29.449	35.096	29.757
b (Å)	5.535	5.210	5.523	5.631
c (Å)	4.132	4.151	4.155	4.306

α (°)	90	90	90	90
β (°)	90	90	90	90
γ (°)	90	90	90	90
V (Å^3)	785.65	636.88	805.39	721.52
R_p, R_{wp},	20.34, 28.96	7.55, 10.42	11.28, 14.80	10.44, 14.46
GOOF	4.29	1.08	1.60	1.78

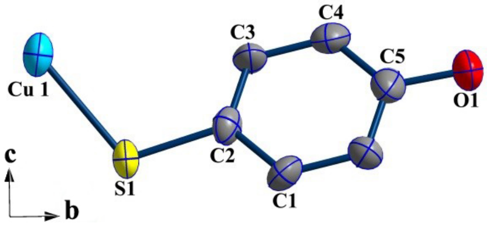

Supplementary Figure 1. Asymmetric structure of Cu(SPh-OH). X-ray single-crystal diffraction indicates that the parent Cu(SPh-OH) crystallizes in the orthorhombic space group Pmn2$_1$ and forms a 2D layer. Herein, the skeleton structure of Cu(SPh-OH) consists of a simple unit [HO-C_6H_4-(μ_3-S)Cu] (Supplementary Fig. 1) which is derived from a μ_3-bridging HO-C_6H_4-SH and three Cu(I). Cu(I) ions are simultaneously coordinated with three S. The most remarkable feature in Cu(SPh-OH) is that Cu ions are linked through S atoms giving rise to a {CuS} graphene-like layer, which is rarely discovered in coordination chemistry.

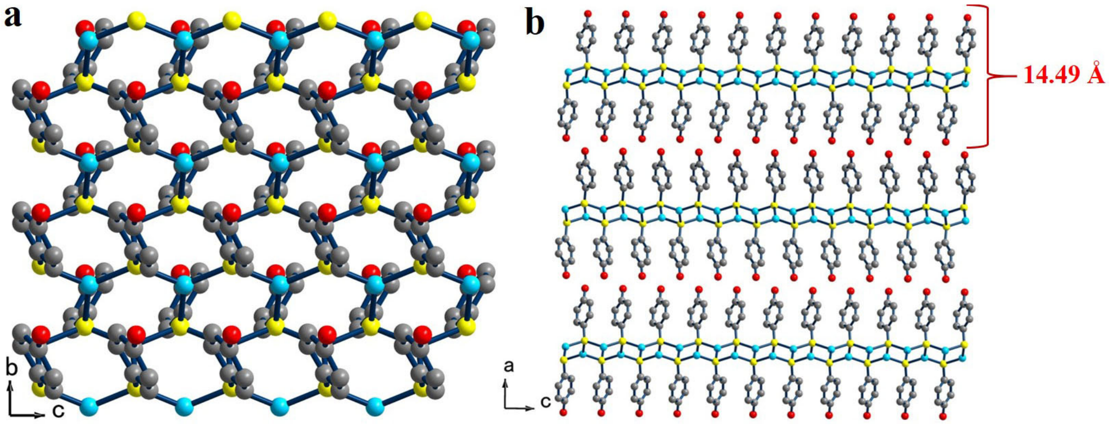

Supplementary Figure 2. a, 2D structure of Cu(SPh-OH) viewed along *a* axis; b, Stacking mode of Cu(SPh-OH) structure. The thickness of the single layer is about 1.45 nm**.**

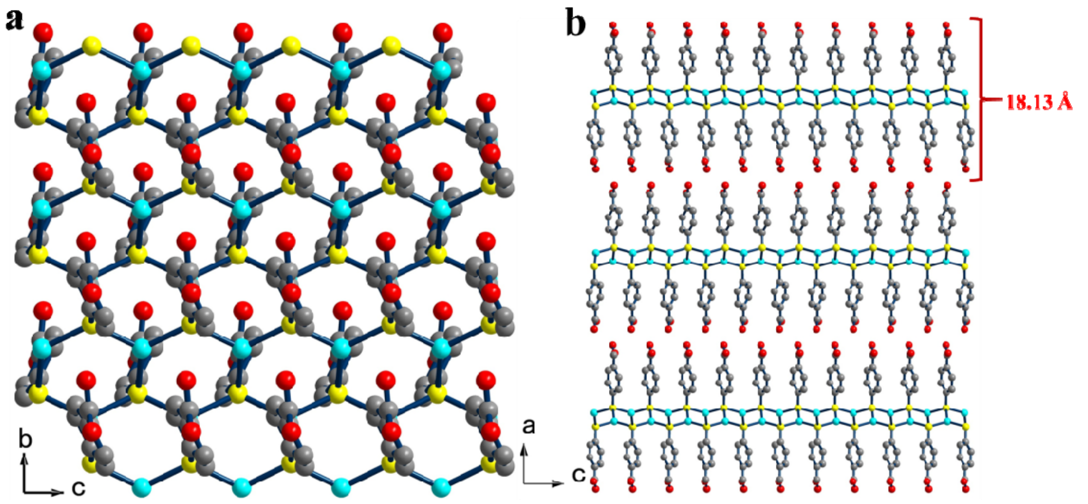

Supplementary Figure 3. a, 2D structure of Cu(SPh-COOH) viewed along *a* axis; b, Stacking mode of Cu(SPh-COOH) structure. The thickness of the single layer is about 1.81 nm.

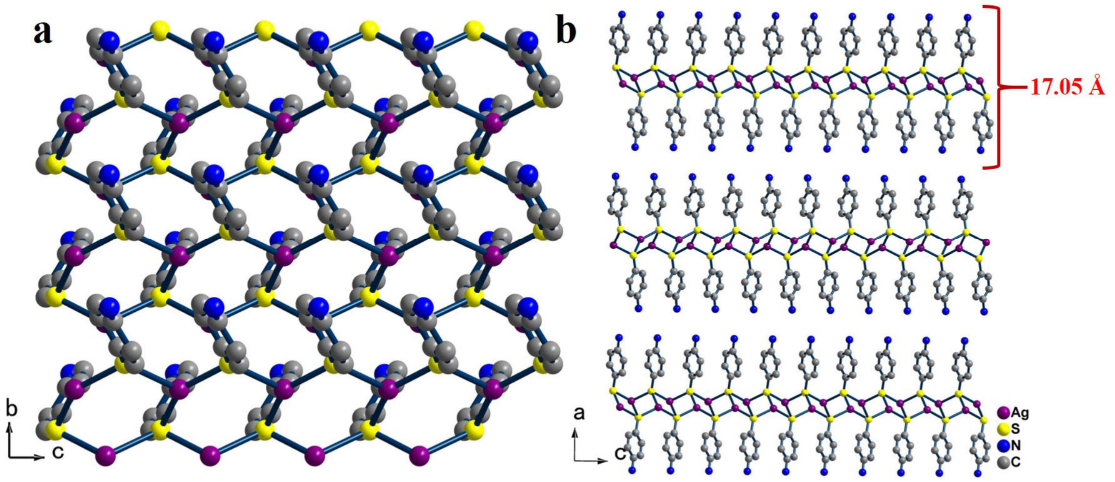

Supplementary Figure 4. a, 2D structure of Ag(SPh-NH_2) viewed along *a* axis; b, Stacking mode of Ag(SPh-NH_2) structure**.** The thickness of the single layer is about 1.71 nm.

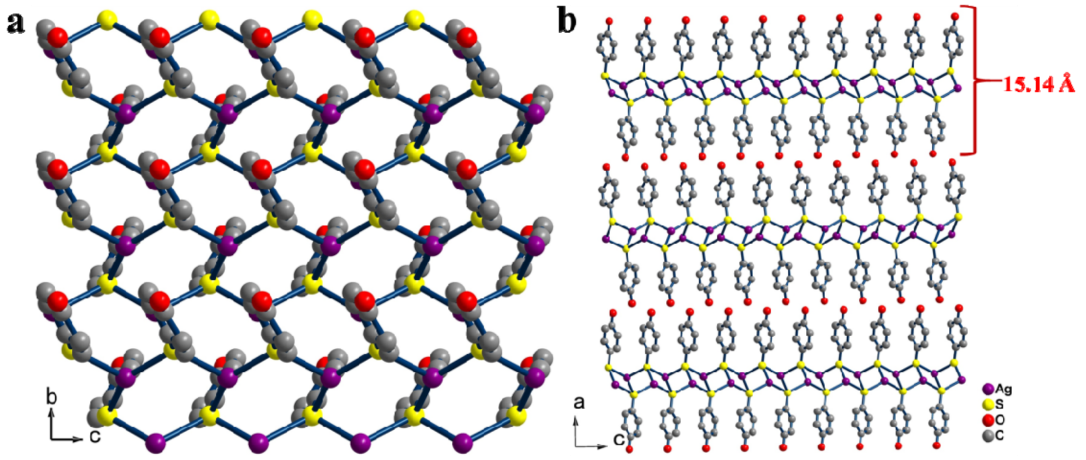

Supplementary Figure 5. a, 2D structure of Ag(SPh-OH) viewed along *a* axis; b, Stacking mode of Ag(SPh-OH) structure. The thickness of the single layer is about 1.51 nm.

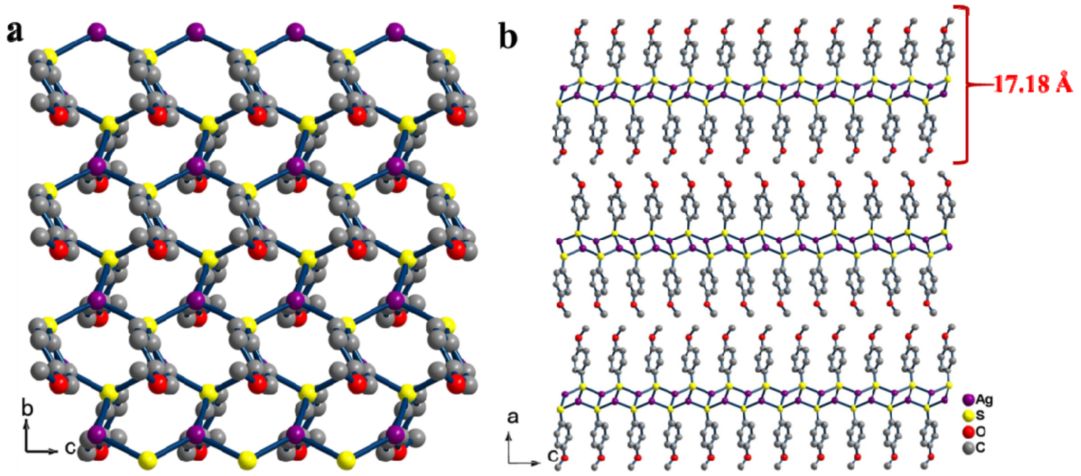

Supplementary Figure 6. a, 2D structure of Ag(SPh-OMe) viewed along *a* axis; b, stacking mode of Ag(SPh-OMe) structure**.** The thickness of the single layer is about 1.72 nm.

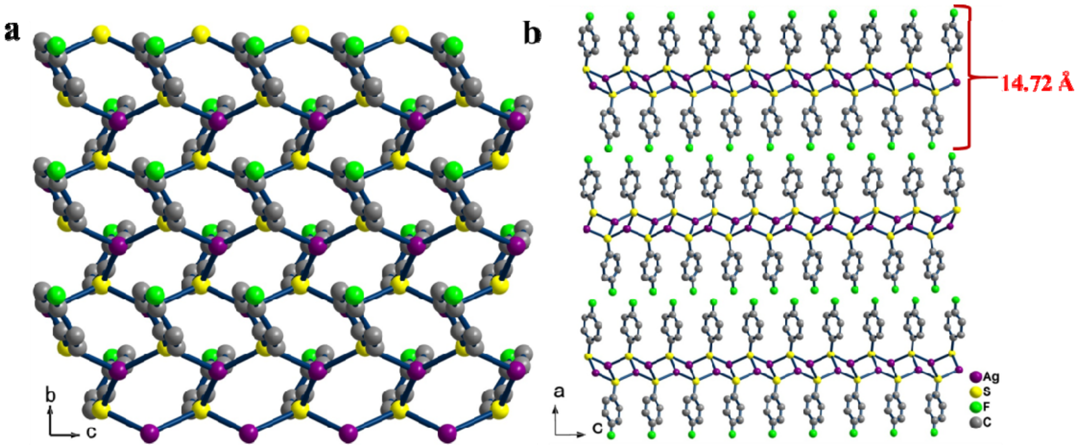

Supplementary Figure 7. 2D structure of Ag(SPh-F) viewed along *a* axis; b, stacking mode of Ag(SPh-F) structure. The thickness of the single layer is about 1.47 nm.

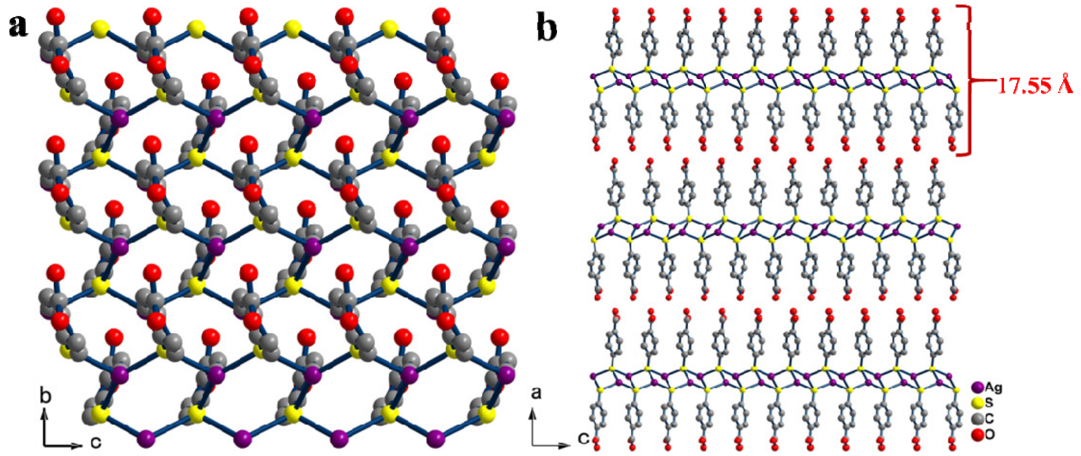

Supplementary Figure 8. 2D structure of Ag(SPh-COOH) viewed along *a* axis; b, Stacking mode of Ag(SPh-COOH) structure. The thickness of the single layer is about 1.76 nm.

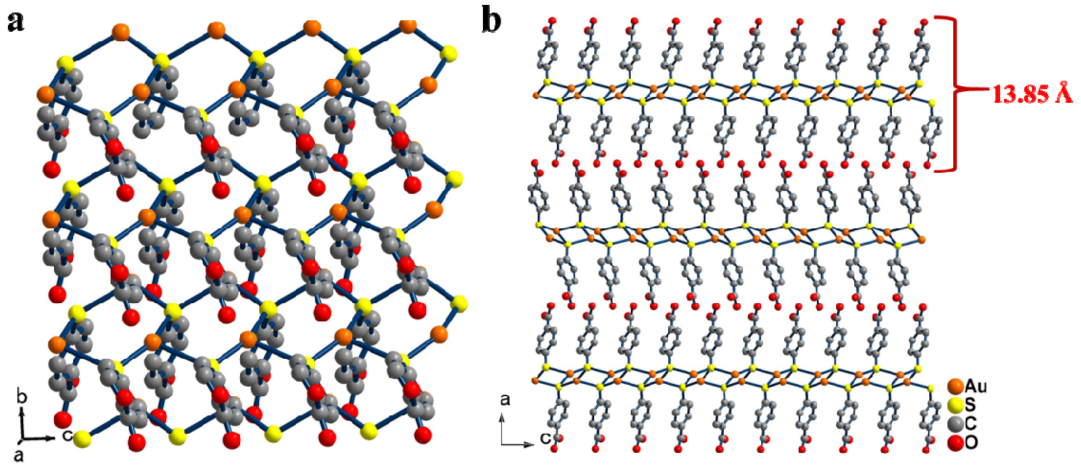

Supplementary Figure 9. 2D structure of Au(SPh-COOH) viewed along *a* axis; b, Stacking mode of Au(SPh-COOH) structure. The thickness of the single layer is about 1.38 nm.

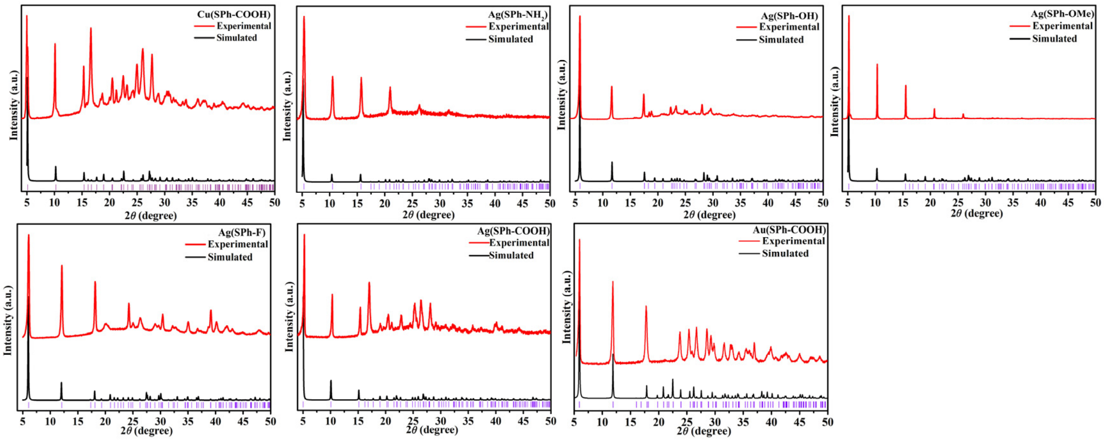

Supplementary Figure 10. Experimental and simulated PXRDs of Cu(SPh-COOH), Ag(SPh-NH_2), Ag(SPh-OH), Ag(SPh-OMe), Ag(SPh-F), Ag(SPh-COOH) and Au(SPh-COOH).

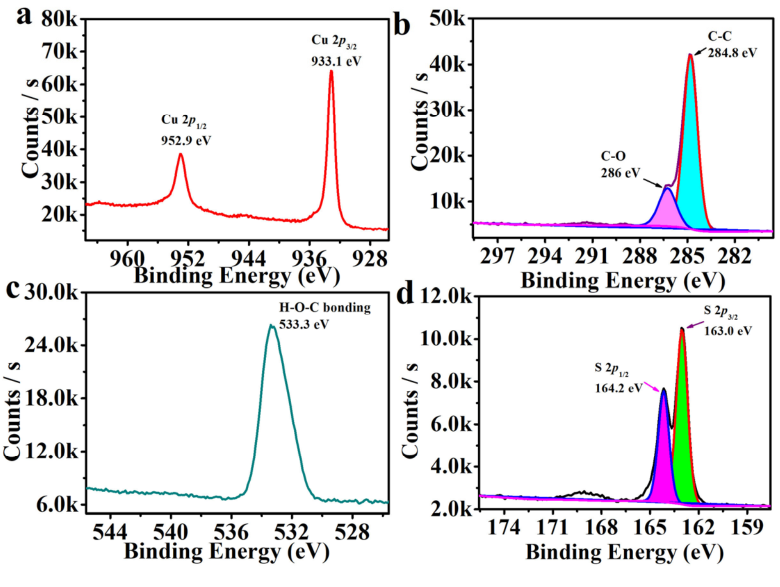

Supplementary Figure 11. XPS spectra of Cu(SPh-OH). a. Cu 2*p* region for Cu(SPh-OH); b. C 1*s* region for Cu(SPh-OH); c. O 1*s* region for Cu(SPh-OH); d. S 2*p* region for Cu(SPh-OH). On

the high binding energy side of the Cu 2$p_{3/2}$ peak, the presence of a Cu 2$p_{3/2}$ component near 933.3 eV indicates the valence of Cu in Cu(SPh-OH) is mono-valence.

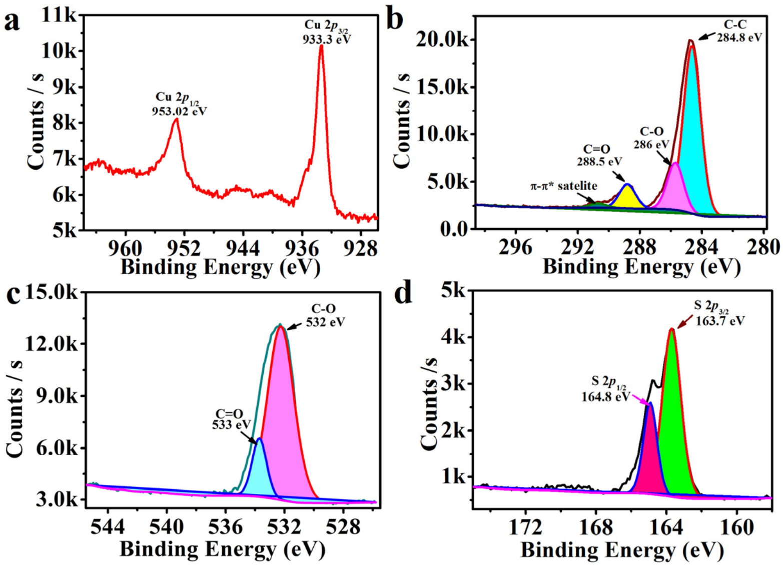

Supplementary Figure 12. XPS spectra of Cu(SPh-COOH). a. Cu 2*p* region for Cu(SPh-COOH); b. C 1*s* region for Cu(SPh-COOH); c. O 1*s* region for Cu(SPh-COOH); d. S 2*p* region for Cu(SPh-COOH). The presence of Cu 2$p_{3/2}$ component at 933.3 eV indicates the valence of Cu in Cu(SPh-COOH) is mono-valence.

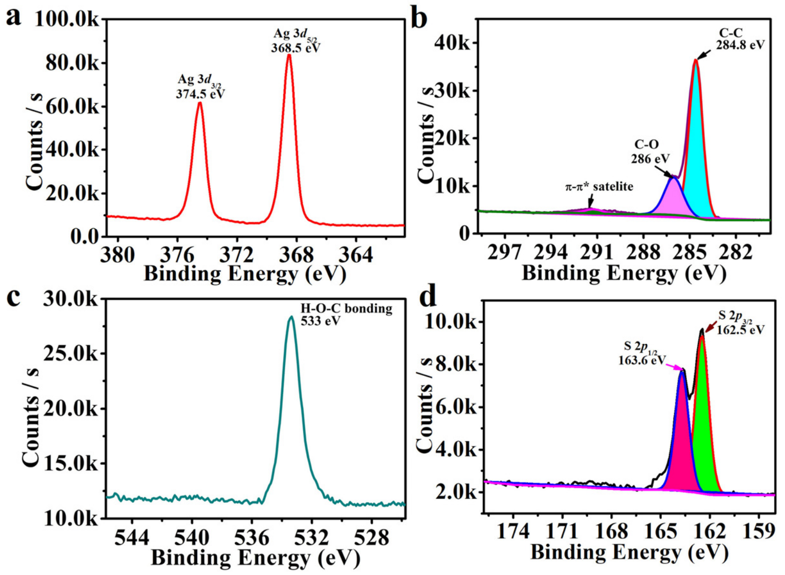

Supplementary Figure 13. XPS spectra of Ag(SPh-OH). a. Ag 3*d* region for Ag(SPh-OH); b. C 1s region for Ag(SPh-OH); c. O 1*s* region for Ag(SPh-OH); d. S 2*p* region for Ag(SPh-OH). Ag 3*d* spectrum of it has two obvious peaks which correspond to Ag 3$d_{5/2}$ at 368.5 eV and Ag 3$d_{3/2}$ at 374.5 eV. The silver in the compound is monovalence.

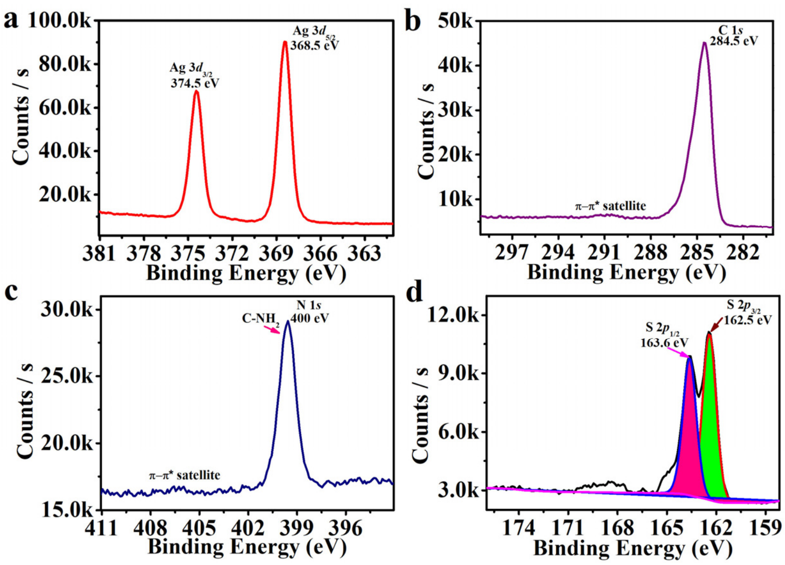

Supplementary Figure 14. XPS spectra of Ag(SPh-NH$_2$). a. Ag 3*d* region for Ag(SPh-NH$_2$); b. C 1*s* region for Ag(SPh-NH$_2$); c. N 1*s* region for Ag(SPh-NH$_2$); d. S 2*p* region for Ag(SPh-NH$_2$). Ag 3*d* spectrum of it has two obvious peaks which correspond to Ag 3$d_{5/2}$ at 368.5 eV and Ag 3$d_{3/2}$ at 374.5 eV**.** The silver in the compound is monovalence.

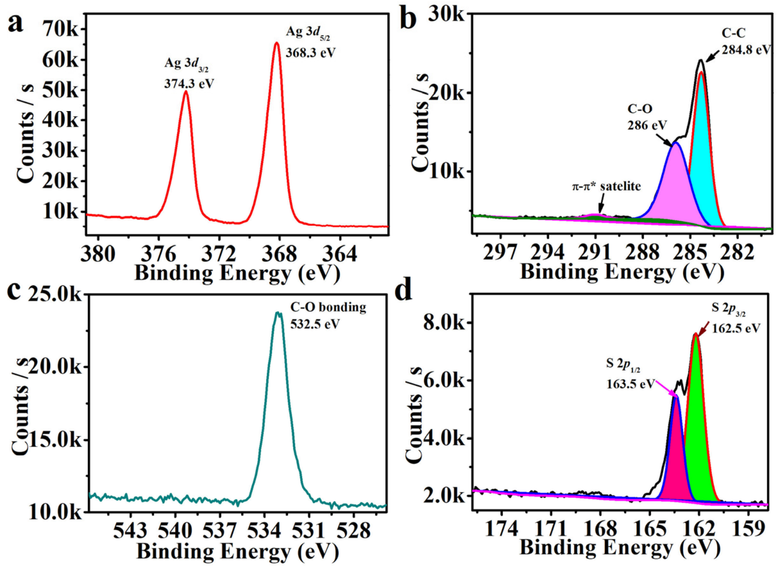

Supplementary Figure 15. XPS spectra of Ag(SPh-OMe). a. Ag 3*d* region for Ag(SPh-OMe); b. C 1*s* region for Ag(SPh-OMe); c. O 1*s* region for Ag(SPh-OMe); d. S 2*p* region for Ag(SPh-OMe). Ag 3*d* spectrum of it has two obvious peaks which correspond to Ag 3$d_{5/2}$ at 368.3 eV and Ag 3$d_{3/2}$ at 374.3 eV, which indicates that the silver in the compound is mono-valance.

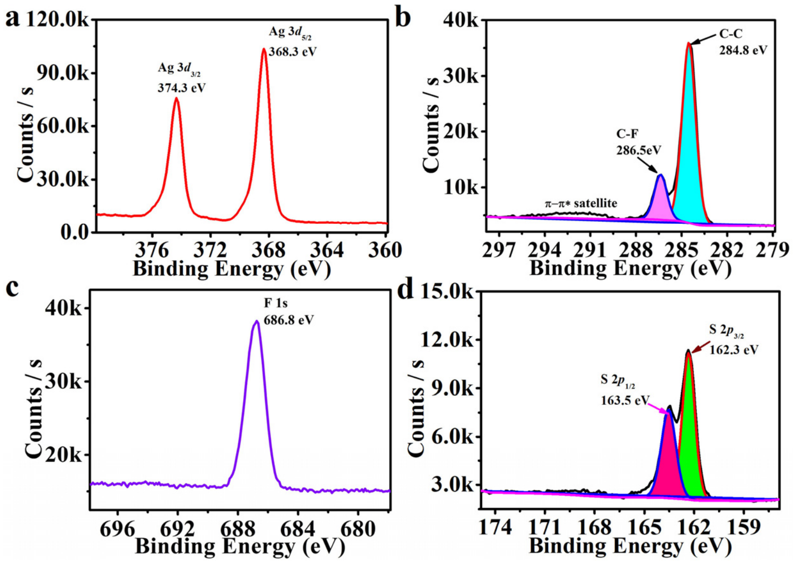

Supplementary Figure 16. XPS spectra of Ag(SPh-F). a. Ag 3*d* region for Ag(SPh-F); b. C 1*s* region for Ag(SPh-F); c. F 1*s* region for Ag(SPh-F); d. S 2*p* region for Ag(SPh-F). Ag 3*d* spectrum of it has two obvious peaks which correspond to Ag $3d_{5/2}$ at 368.3 eV and Ag $3d_{3/2}$ at 374.3 eV**.** The silver in the compounds is monovalence.

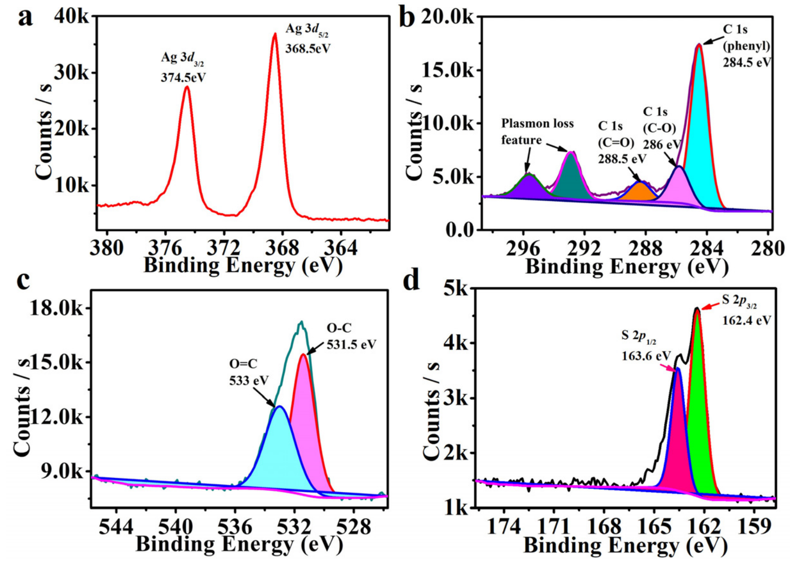

Supplementary Figure 17. XPS of Ag(SPh-COOH). a. Ag 3*d* region for Ag(SPh-COOH); b. C 1*s* region for Ag(SPh-COOH); c. O 1*s* region for Ag(SPh-COOH); d. S 2*p* region for Ag(SPh-COOH). Ag 3*d* spectrum of it has two obvious peaks which correspond to Ag $3d_{5/2}$ at 368.5 eV and Ag $3d_{3/2}$ at 374.5 eV**.** The silver in the compound is monovalence.

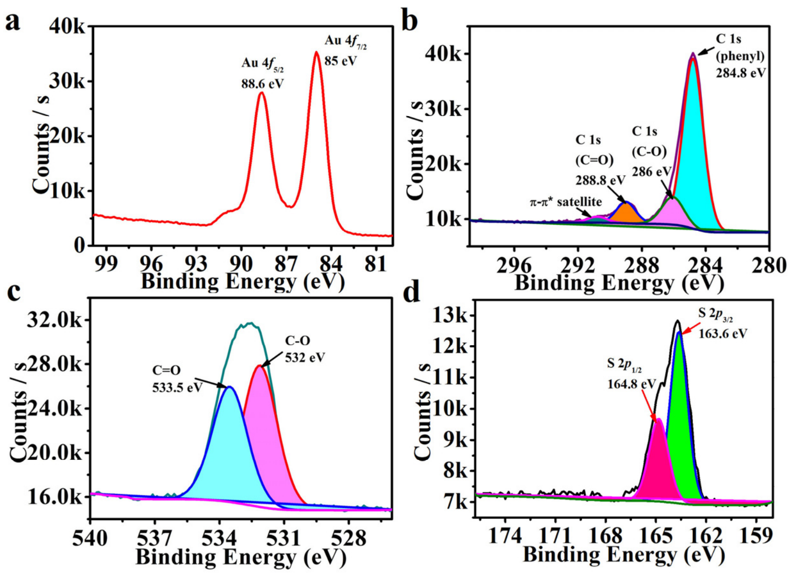

Supplementary Figure 18. XPS of Au(SPh-COOH). a. Ag 3*d* region for Au(SPh-COOH); b. C 1*s* region for Au(SPh-COOH); c. O 1*s* region for Au(SPh-COOH); d. S 2*p* region for Au(SPh-COOH). The peaks at the binding energy 85.0 eV for Au 4$f_{7/2}$ and 88.6 eV for Au 4$f_{5/2}$ are derived from the mono-valence Au atom.

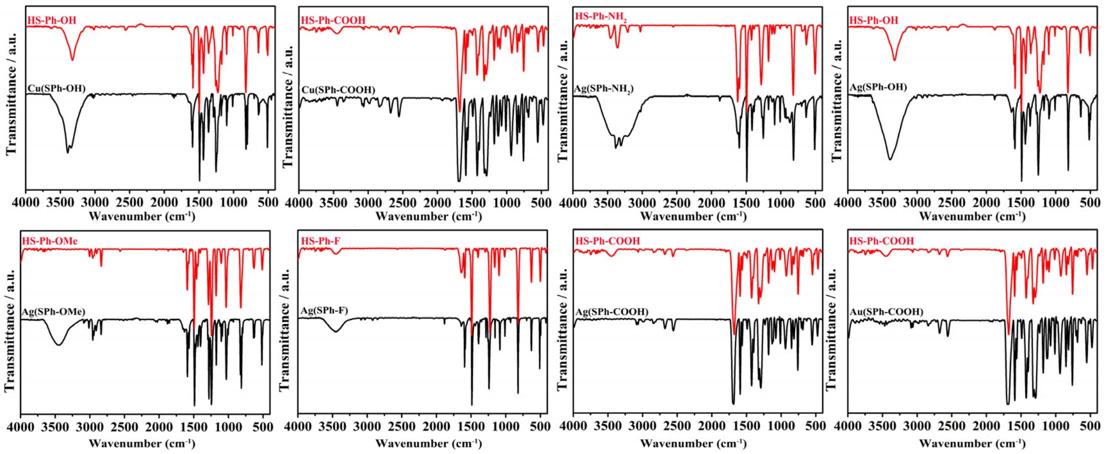

Supplementary Figure 19. FT-IR spectra of OMCs. The vibration bands of Ph unit at 1400–1590 cm^{-1} can be observed in all OMCs. In Cu(SPh-OH) and Ag(SPh-OH), there are ν(OH) bands at 3250–3750 cm^{-1}. Meanwhile, for Cu(SPh-COOH), Ag(SPh-COOH), and Au(SPh-COOH): antisymmetric vibrations of C=O is present at about 1685 cm^{-1}. For Ag(SPh-NH_2), two characteristic peaks at 3300–3500 cm^{-1} are the stretching vibration of –NH_2. For Ag(SPh-OMe), the characteristic peak of –CH_3 stretching is present at 2870–3005 cm^{-1}. For Ag(SPh-F), the strong peak at 1237 cm^{-1} belongs to the stretching vibration of C–F.

Supplementary Table 5. Elemental Analysis results of the series of compounds

Formula	Logogram	C (wt %)		H (wt %)		S (wt %)	
		E[a]	T[b]	E[a]	T[b]	E[a]	T[b]
C_6H_5CuOS	Cu(SPh-OH)	38.15	38.18	2.48	2.67	17.41	16.99
$C_7H_5CuO_2S$	Cu(SPh-COOH)	38.68	38.79	2.77	2.33	14.80	15.01
C_6H_6AgNS	Ag(SPh-NH_2)	30.81	31.05	2.81	2.61	13.82	13.82

C_6H_5AgOS	Ag(SPh-OH)	30.91	30.92	2.08	2.16	13.77	13.76
C_7H_7AgOS	Ag(SPh-OMe)	33.54	34.02	2.61	2.86	13.35	12.98
C_6H_4AgFS	Ag(SPh-F)	30.89	30.66	1.54	1.72	14.00	13.64
$C_7H_5AgO_2S$	Ag(SPh-COOH)	32.23	32.21	1.84	1.93	13.02	12.28
$C_7H_5AuO_2S$	Au(SPh-COOH)	24.80	24.01	1.42	1.44	9.44	9.16

[a] Experimental results. [b] Theoretical value.

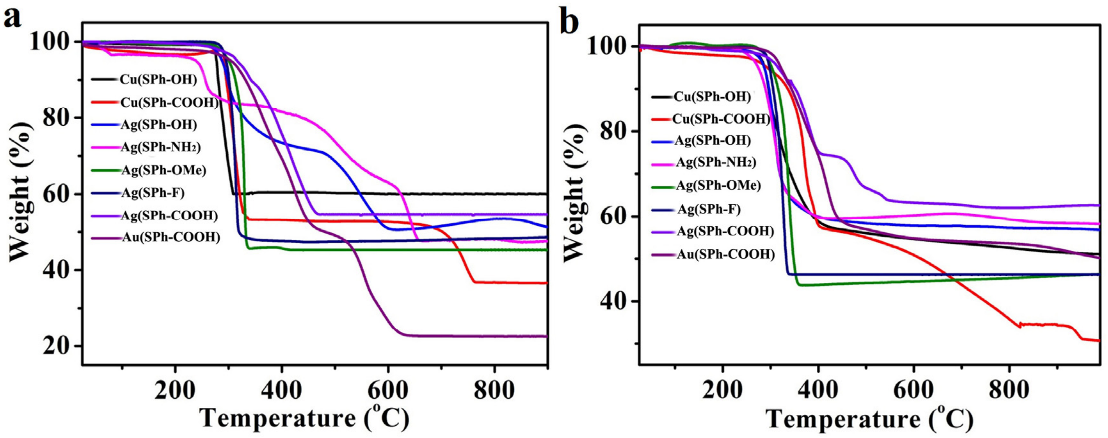

Supplementary Figure 20. TGA trace of OMCs under (a) air atmosphere and (b) nitrogen flow. In air atmosphere, Cu(SPh-COOH), Ag(SPh-OH), Ag(SPh-NH_2) and Au(SPh-COOH) have two weight loss stages, while the compounds Cu(SPh-OH), Ag(SPh-OMe), Ag(SPh-F) and Ag(SPh-COOH) showed only one obvious weight loss stage in the range of 270 to 900 ºC, which are attribute to the decomposition of structure. In nitrogen atmosphere condition, except Ag(SPh-COOH), all the other compounds, has only one obvious weight loss stage starting at 280 ºC, which is the onset temperature for the gradual thermal decomposition. While Ag(SPh-COOH) showed obviously two step weight loss.

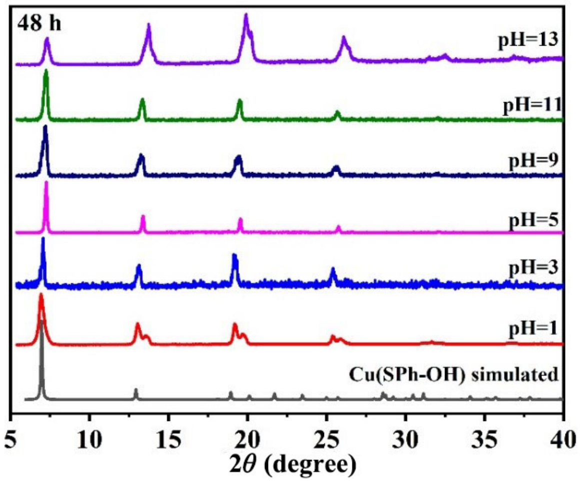

Supplementary Figure 21. PXRD of Cu(SPh-OH) in different pH solution for 48 hours.

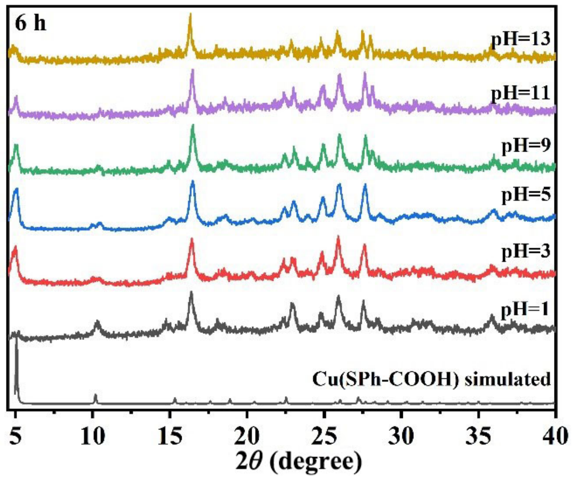

Supplementary Figure 22. PXRD of Cu(SPh-COOH) in different pH solution for 6 hours.

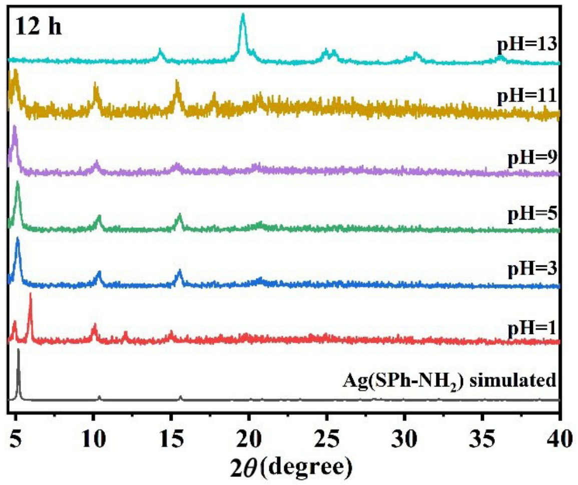

Supplementary Figure 23. PXRD of Ag(SPh-NH_2) in different pH solution.

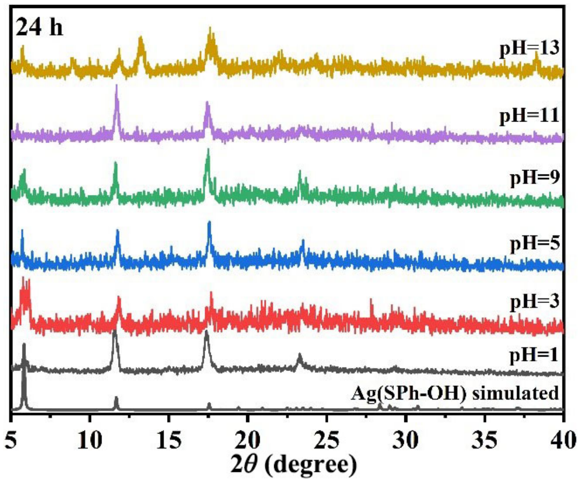

Supplementary Figure 24. PXRD of Ag(SPh-OH) in different pH solution for 24 hours.

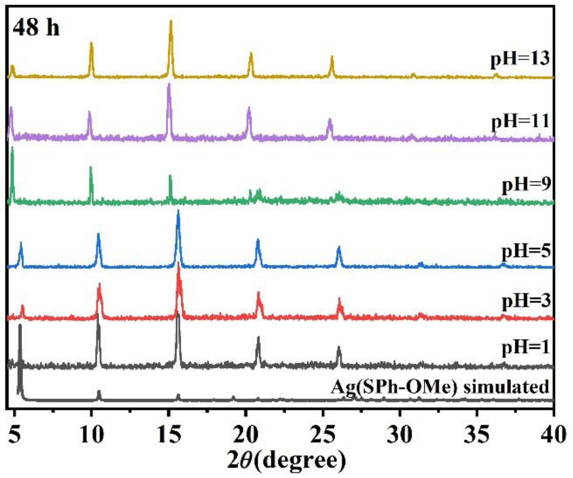

Supplementary Figure 25. PXRD of Ag(SPh-OMe) in different pH solution for 48 hours.

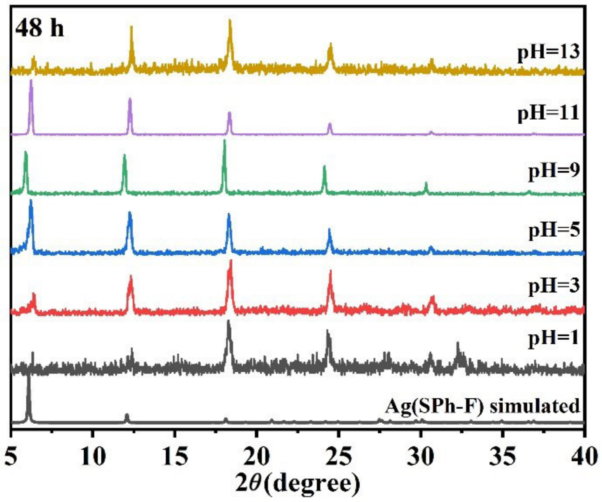

Supplementary Figure 26. PXRD of Ag(SPh-F) in different pH solution for 48 hours.

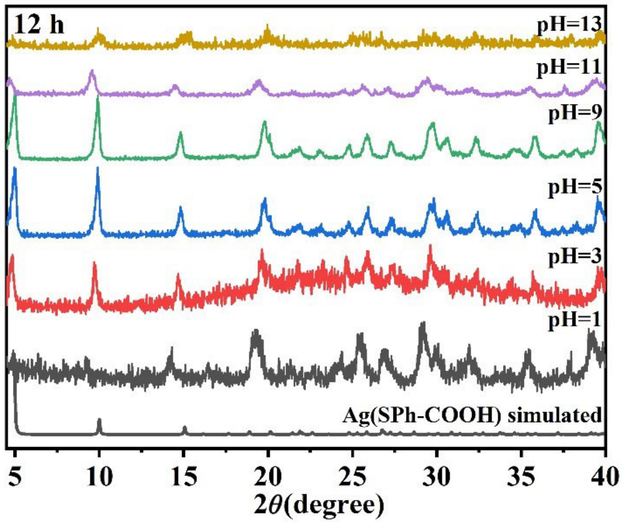

Supplementary Figure 27. PXRD of Ag(SPh-COOH) in different pH solution for 12 hours.

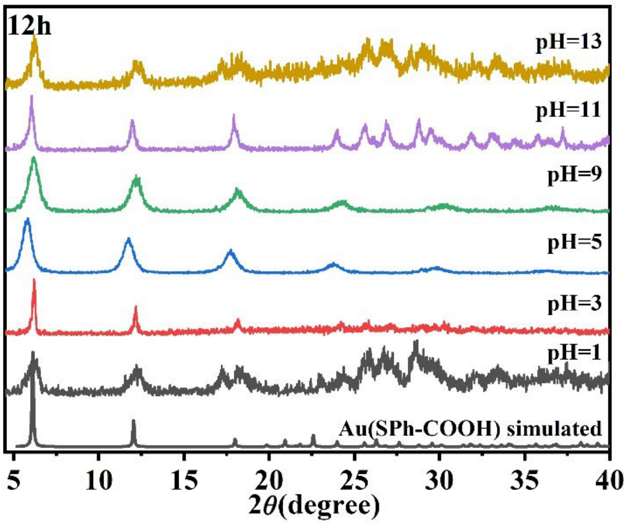

Supplementary Figure 28. PXRD of Au(SPh-COOH) in different pH solution for 12 hours.

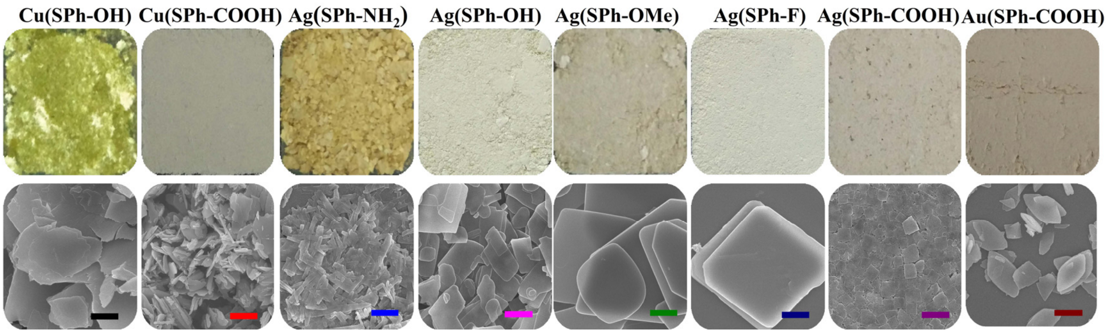

Supplementary Figure 29. Photographs and SEM images of OMCs. Scale bar: 2 μm.

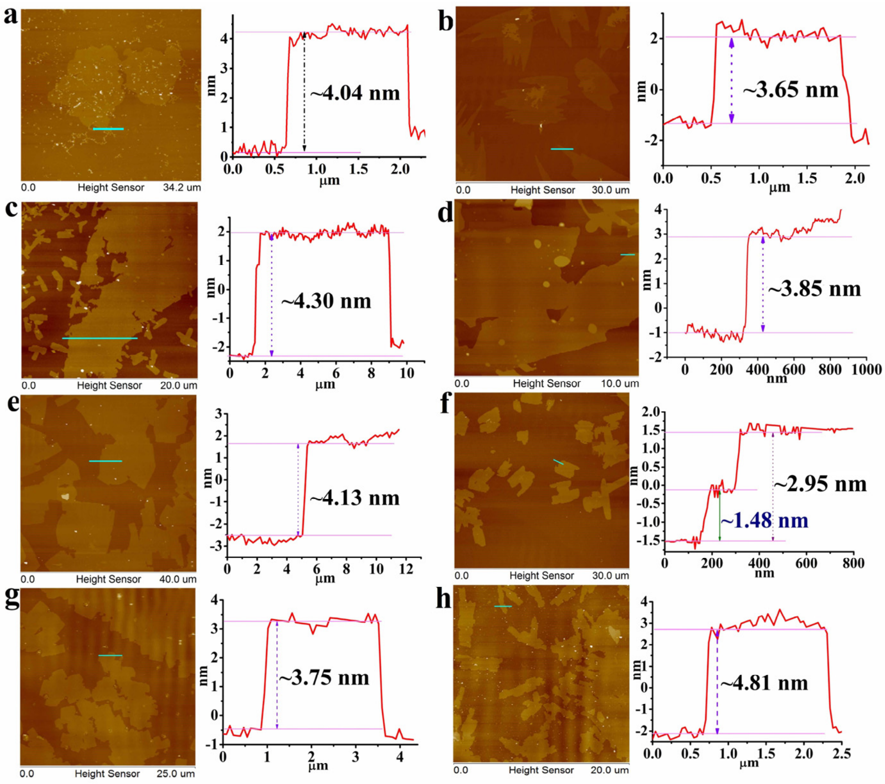

Supplementary Figure 30. AFM images of (**a**) Cu(SPh-OH), (**b**) Cu(SPh-COOH), (**c**) Ag(SPh-NH_2), (**d**) Ag(SPh-OH), (**e**) Ag(SPh-OMe), (**f**) Ag(SPh-F), (**g**) Ag(SPh-COOH) and (**h**) Au(SPh-COOH) on SiO_2/Si substrates.

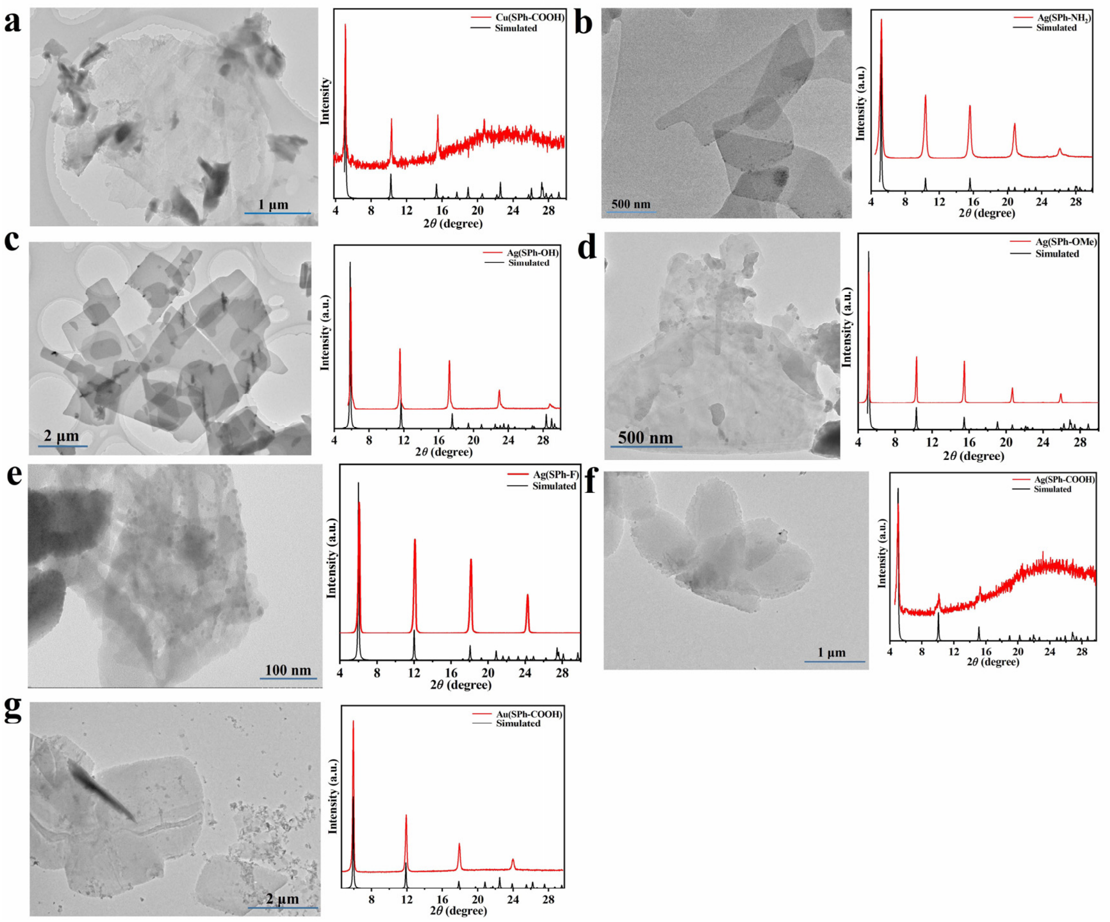

Supplementary Figure 31. TEM images and their corresponding XRD patterns of OMCs.

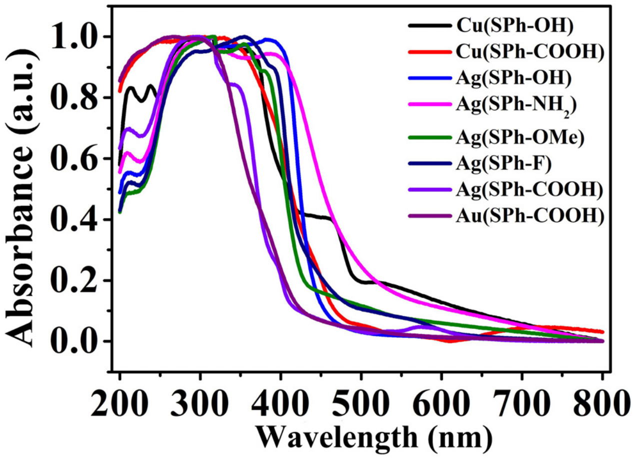

Supplementary Figure 32. Solid state UV-Vis adsorption spectra of OMCs**.**

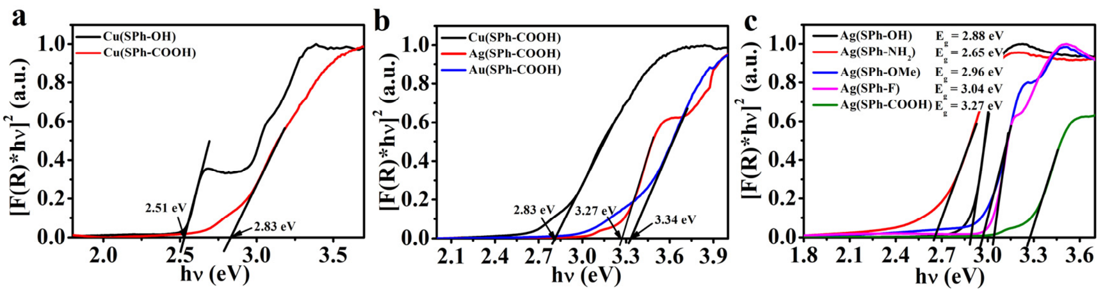

Supplementary Figure 33. Kubelka–Munk elaboration for (**a**) Cu(SPh-OH) and Cu(SPh-COOH); (**b**) Cu(SPh-COOH), Ag(SPh-COOH) and Au(SPh-COOH); (**c**) Ag(SPh-OH), Ag(SPh-NH_2), Ag(SPh-OMe), Ag(SPh-F) and Ag(SPh-COOH)**.**

UV-Vis diffused reflectance spectra of OMCs in the wavelength range from 200 to 800 nm are shown in Supplementary Fig. 33. Experimental data of diffuse reflectance were converted to absorption coefficient values F(R) according to the Kubelka–Munk equation:

$$F(R_\infty) = \frac{(1 - R_\infty)^2}{2R_\infty} \tag{1}$$

Where $F(R_\infty)$ ($R_\infty = R_{sample}/R_{standard}$) is the absorption coefficient.

The theory of the interband optical absorption shows that at the absorption edge, the absorption coefficient of a semiconductor can be expressed as：

$$[F(R_\infty)h\nu]^n = A(h\nu - E_g) \tag{2}$$

where A is a constant, E_g is the band-gap of allowed transitions (eV), h is the Planck's constant (6.626×10^{-34} J s), ν is the frequency of the light (s^{-1}), and the exponent n is a number characterizing the transition process, which usually depends upon the transition type, n = 2, 2/3, 1/2, and 1/3 denotes to allowed direct, forbidden direct, indirect and forbidden indirect transitions, respectively. From the analysis, we take n = 2 (direct) for OMCs. Therefore, a transformed Kubelka–Munk function can be constructed by plotting $[F(R_\infty)]^2$ against the energy of excitation source to obtain the band-gap of OMCs. The convert curves were fitted by a Boltzman sigmoidal equation and the band gap values were obtained by the x-axis intercept of the line tangent to the inflection point of the curve. Supplementary Fig. 33 reports the transformed reflectance as a function of the photon energy for the OMCs. The values show a progressive change with different of metal ions and substituent groups of the ligands.

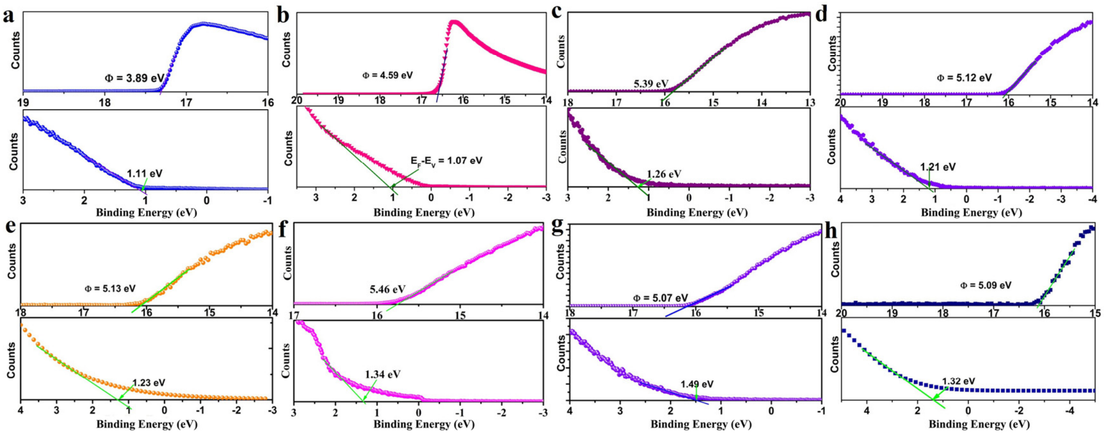

Supplementary Figure 34. UPS data of (**a**) Cu(SPh-OH), (**b**) Cu(SPh-COOH), (**c**) Ag(SPh-NH_2), (**d**) Ag(SPh-OH), (**e**) Ag(SPh-OMe), (**f**) Ag(SPh-F), (**g**) Ag(SPh-COOH) and (**h**) Au(SPh-COOH).

The work function (*Φ*) and the difference between Fermi level and valence band (E_F-E_v) of OMCs can be extracted from ultraviolet photoelectron spectroscopy (UPS) measurement (light source He I, 21.22 eV). The UPS signal at the higher binding energy corresponds to the photoemission cut-off from which Φ can be extracted. The UPS onset at the lower binding energy corresponds to electrons photo-emitted from the top of the filled state, from which the difference between Fermi level and valence band (E_F-E_v) can be extracted. For example, for Cu(SPh-OH) in Supplementary Fig. 34a, the work function and E_F-E_V were 3.89 eV and 1.11 eV, respectively. Given its optical band gap of 2.51 eV, the absolute position of the valence-band maxima (E_V) and conduction-band minima (E_C) in Cu(SPh-OH) are calculated to be 5.00 eV and 2.49 eV, respectively, below the vacuum level.

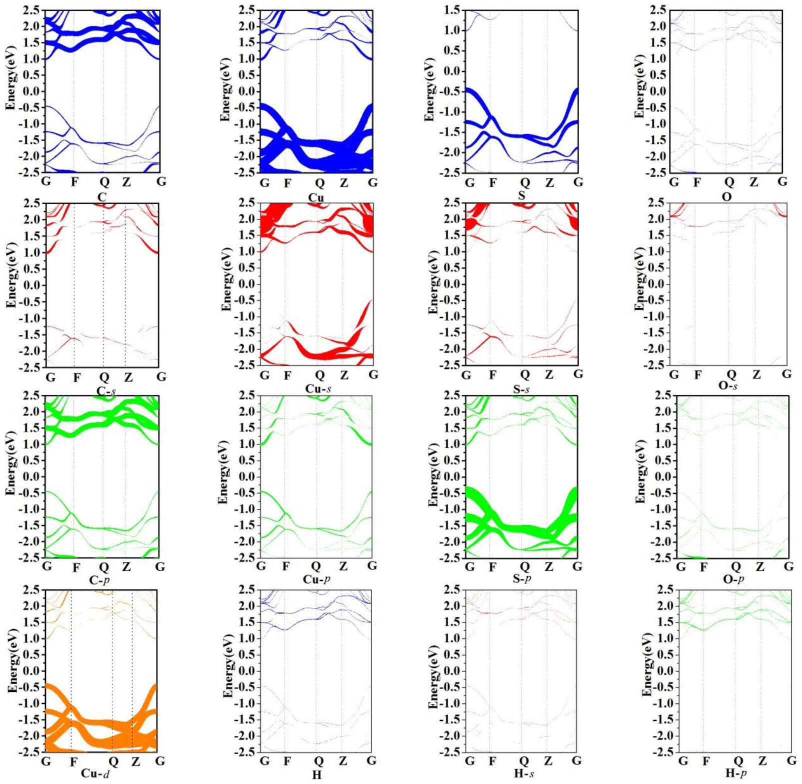

Supplementary Figure 35. More detail information about DFT calculation result of Cu(SPh-OH).

The band structure of Cu(SPh-OH) features a direct bandgap of 1.44 eV. In comparison with Cu(SPh-OH), the band structure of Ag(SPh-OH) features a direct bandgap of 1.95 eV and the band structure of Au(SPh-COOH) features an indirect bandgap of 2.19 eV. The band decomposed charge density at the Gamma (G) point was calculated to check the electronic contribution of each element. As shown in Supplementary Fig. 35-37, for Cu(SPh-OH), Ag(SPh-OH) and Au(SPh-COOH), the VBM/CBM band at the G point are mainly constructed by the M-*d* orbitals and S-*p* orbitals. However, it is also found that the C atom which is directly bonded to S also has little contribution to the VBM/CBM band. The relative magnitudes of their bandgaps (E_{gap}(Au[SPh-COOH]) > E_{gap}(Ag[SPh-OH]) > E_{gap}(Cu[SPh-OH])) agree well with the order of the metals' relative electronegativities, i.e., χ(Au) > χ(Ag) > χ(Cu), which originates from their increasingly localized d orbitals of the IB group elements going down the periodic table.

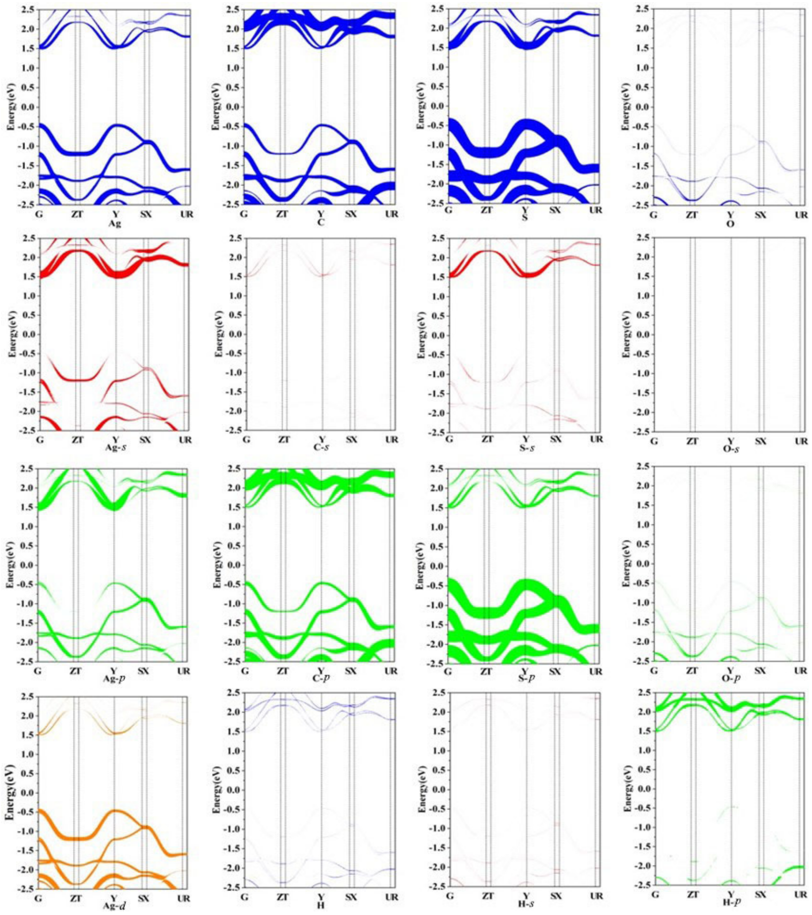

Supplementary Figure 36. DFT calculations results of the electronic contribution from corresponding component elements in Ag(SPh-OH).

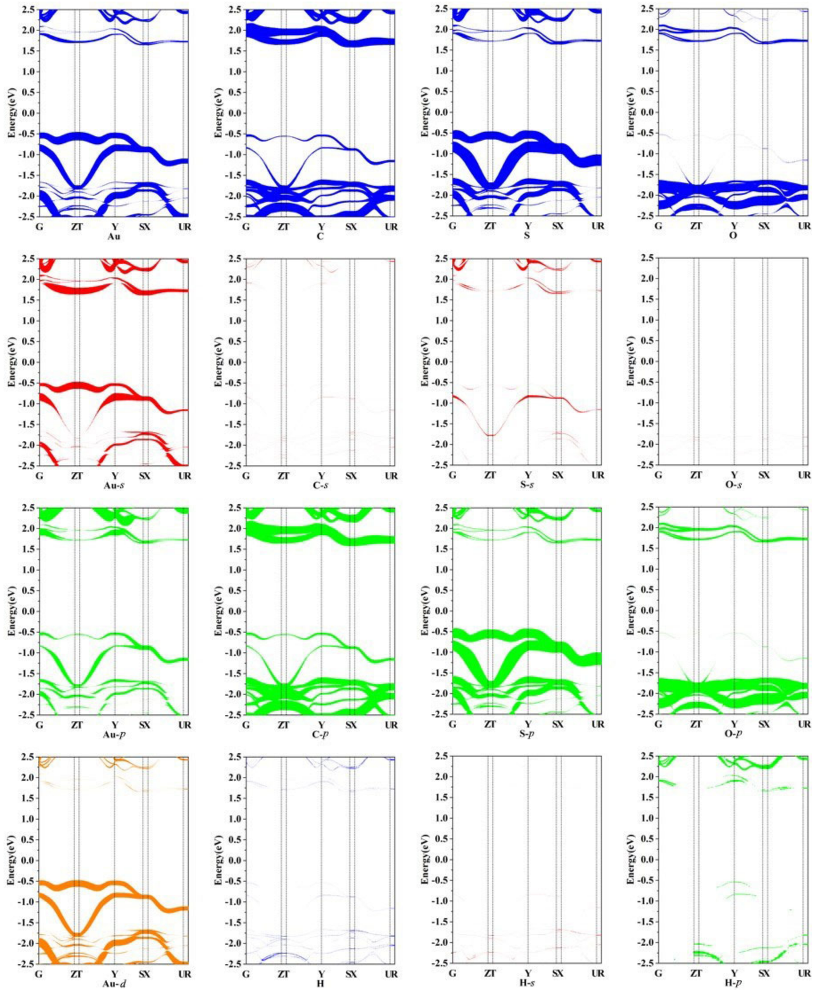

Supplementary Figure 37. DFT calculations results of the electronic contribution from corresponding component elements in Au(SPh-COOH).

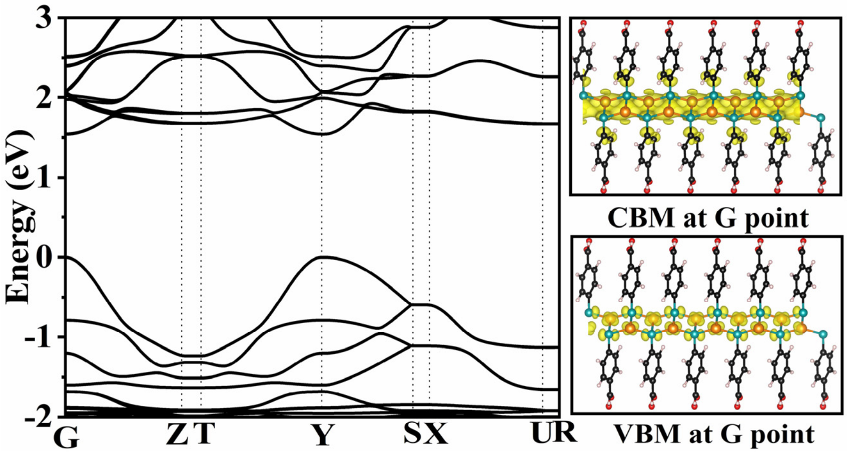

Supplementary Figure 38. Band structure of Cu(SPh-COOH).

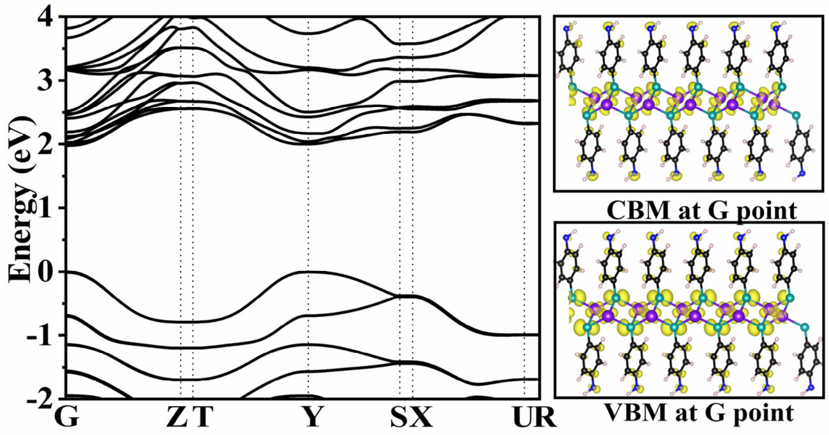

Supplementary Figure 39. Band structure of Ag(SPh-NH_2).

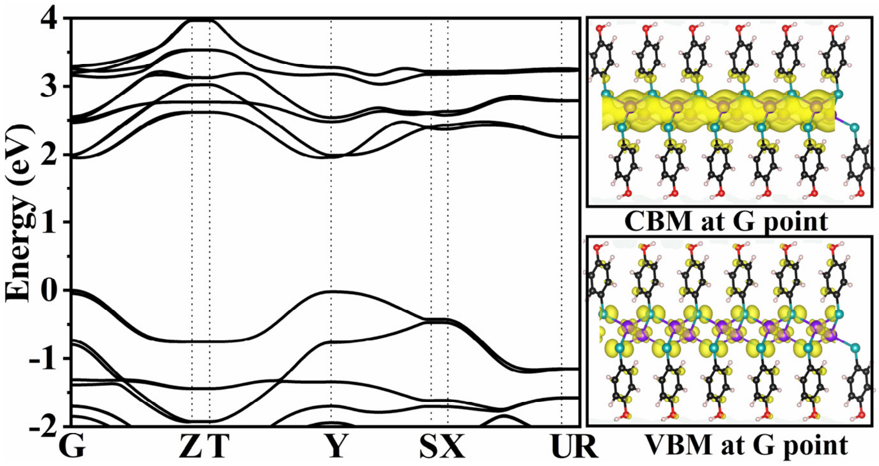

Supplementary Figure 40. Band structure of Ag(SPh-OH).

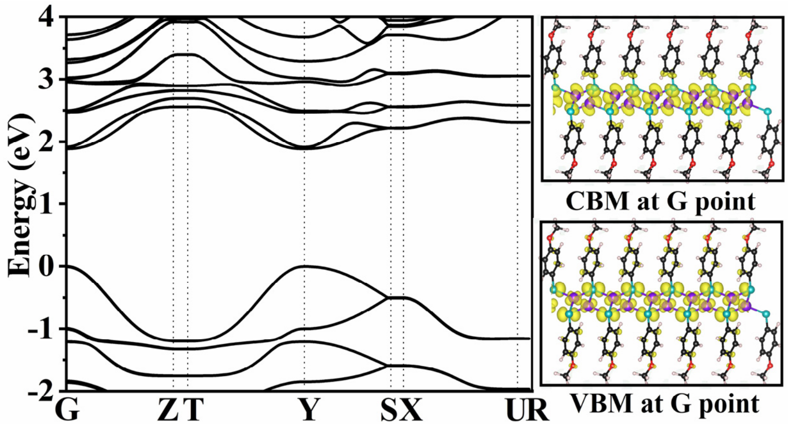

Supplementary Figure 41. Band structure of Ag(SPh-OMe).

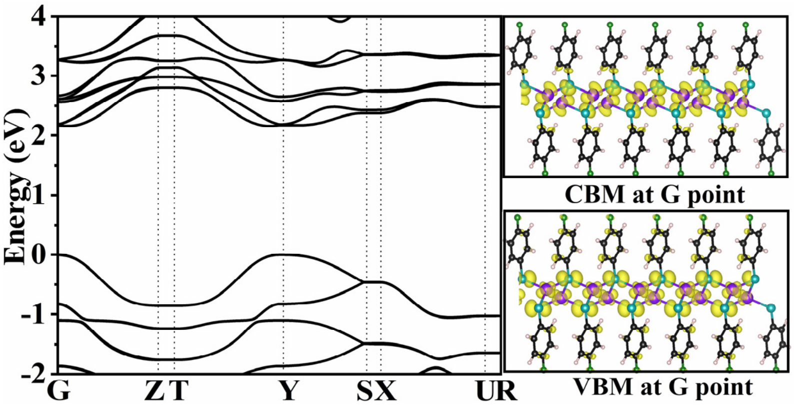

Supplementary Figure 42. Band structure of Ag(SPh-F).

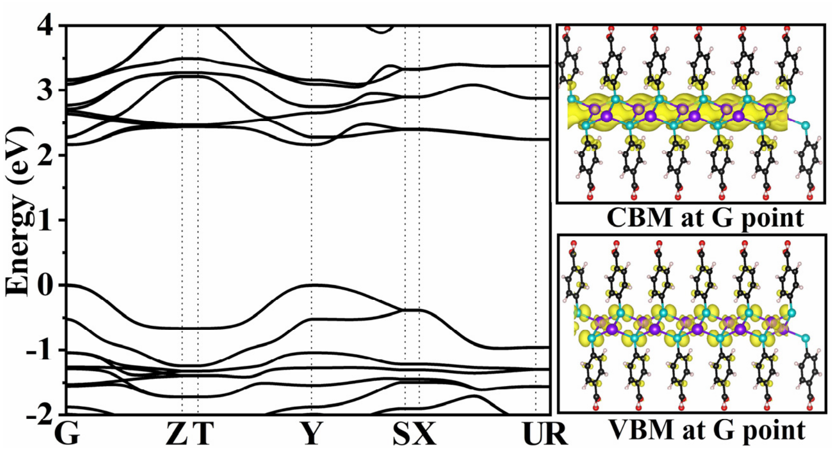

Supplementary Figure 43. Band structure of Ag(SPh-COOH).

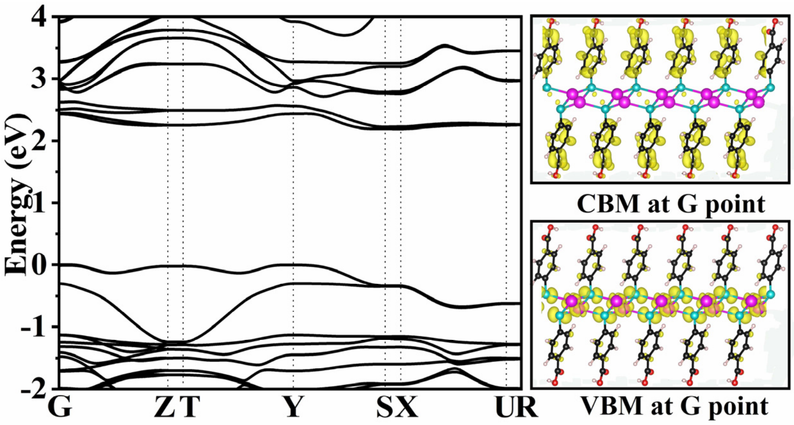

Supplementary Figure 44. Band structure of Au(SPh-COOH).

In addition, we also summaries the calculated and experimental ionization potentials (IPs) and the electron affinities (EAs) of Cu(SPh-OH), Ag(SPh-OH) and Cu(SPh-COOH) with single layer, bilayer and trilayer in Supplementary Table 6 and Supplementary Fig. 45-47. The calculation results of the longitudinal comparison of DFT show that the layer number of OMCs has little effect on IPs and EAs. However, the selection of metals and ligands has a great influence on the electronic properties of the OMCs.

Supplementary Table 6. DFT calculations on the Ionization potentials (IPs) and electron affinities (EAs) for Cu(SPh-OH), Ag(SPh-OH) and Cu(SPh-COOH) with single layer, bilayer and

trilayer; Experimental IPs and EAs (derived from UPS and UV-Vis absorption spectra) of Cu(SPh-OH), Ag(SPh-OH) and Cu(SPh-COOH).

		(eV)	Cu(SPh-OH)	Ag(SPh-OH)	Cu(SPh-COOH)
**Calculation**	**single layer**	IPs	3.72	4.48	5.97
		EAs	2.28	2.50	4.44
	**bilayer**	IPs	3.71	4.60	5.96
		EAs	2.28	2.64	4.43
	**trilayer**	IPs	3.64	4.49	5.98
		EAs	2.23	2.53	4.44
**Experimental**	**Few-layer**	IPs	5.00	6.33	5.66
		EAs	2.49	3.45	2.83

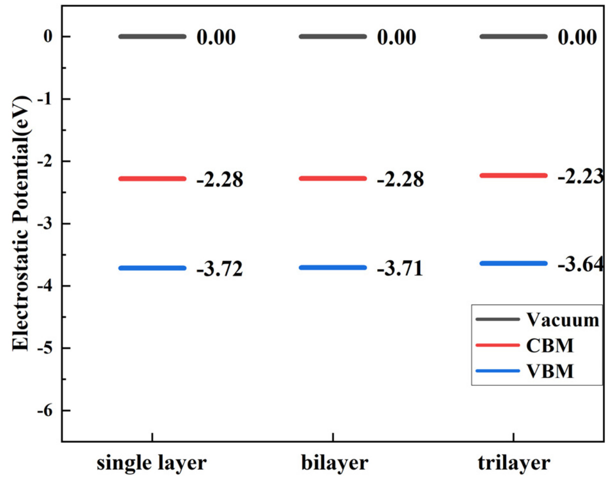

Supplementary Figure 45. DFT calculations on the electrostatic potentials of Cu(SPh-OH) with single layer, bilayer and trilayer.

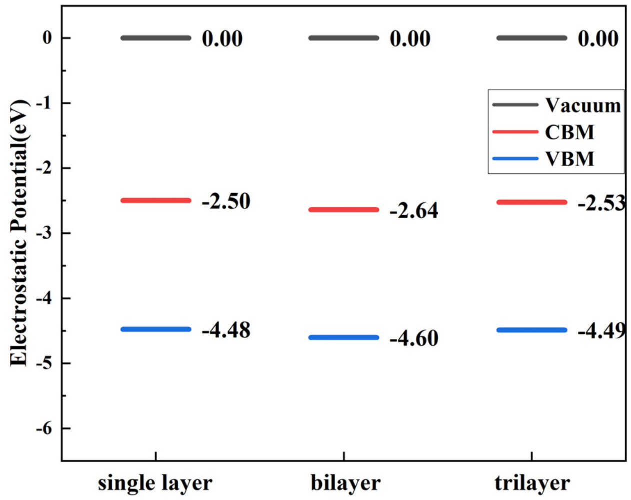

Supplementary Figure 46. DFT calculations on the electrostatic potentials of Ag(SPh-OH) with single layer, bilayer and trilayer.

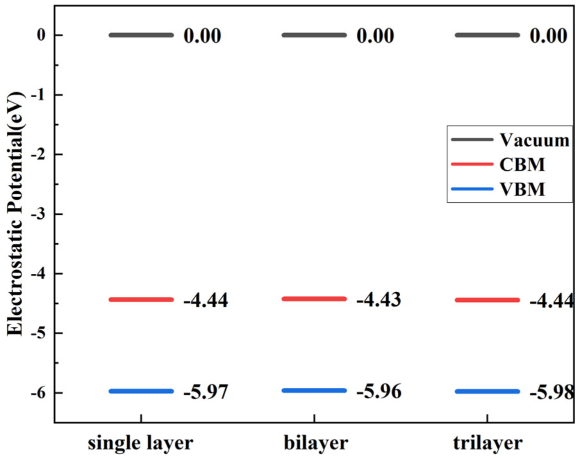

Supplementary Figure 47. DFT calculations on the electrostatic potentials of Cu(SPh-COOH) with single layer, bilayer and trilayer.

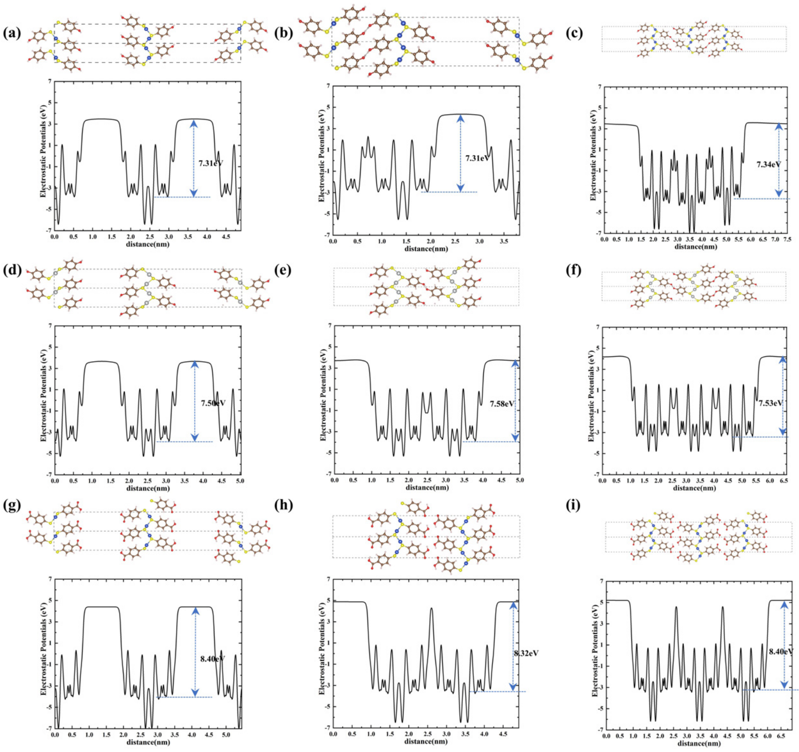

Supplementary Figure 48. DFT calculations on the electrostatic potentials of Cu(SPh-OH) with (a) single layer, (b) bilayer and (c) trilayer; Ag(SPh-OH) with (d) single layer, (e) bilayer and (f) trilayer; Cu(SPh-COOH) with (g) single layer, (h) bilayer and (i) trilayer. It is found that the electrostatic potentials of single layer, bilayer and trilayer of these compounds have no/little shift of the vacuum levels with number of layers. Thus, for few-layer OMCs, their corresponding VBMs and CBMs barely shift.

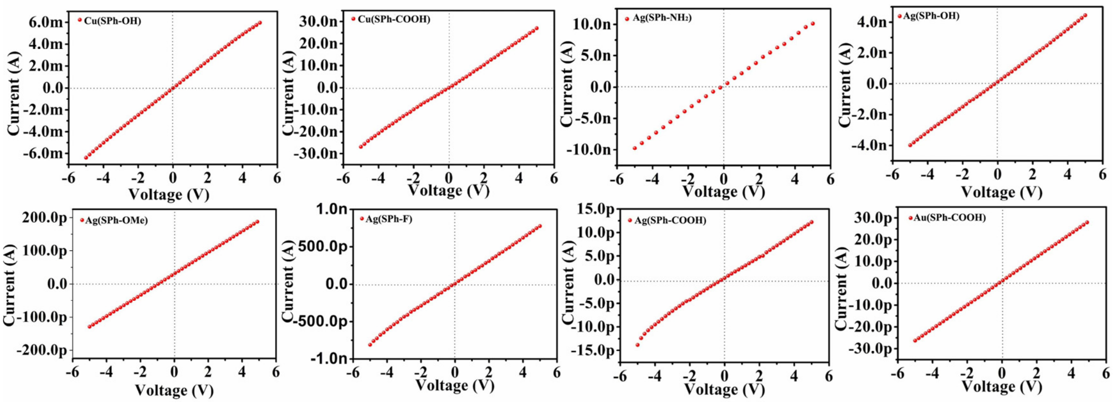

Supplementary Figure 49. Typical I-V curves of OMCs in the voltage range from –5 V to 5 V.

Supplementary Table 7. Conductivity values of OMCs.

**Sample name**	**Average conductivity (S/cm)**	**Conductivity values (S/cm)**			**Standard error**
		**Sample 1**	**Sample 2**	**Sample 3**	
**Cu(SPh-OH)**	2.10×10^{-3}	1.21×10^{-3}	2.55×10^{-3}	2.53×10^{-3}	5.911×10^{-4}
**Cu(SPh-COOH)**	4.58×10^{-9}	5.62×10^{-9}	3.56×10^{-9}	4.57×10^{-9}	6.91×10-^{10}
**Ag(SPh-NH$_2$)**	1.58×10^{-8}	1.11×10^{-8}	1.57×10^{-8}	2.06×10^{-8}	3.2×10^{-9}
**Ag(SPh-OH)**	2.8×10^{-9}	1.58×10^{-9}	3.6×10^{-9}	3.22×10^{-9}	8.13×10^{-10}
**Ag(SPh-OMe)**	5.45×10^{-10}	7.93×10^{-10}	3.32×10^{-10}	5.1×10^{-10}	1.65×10^{-10}
**Ag(SPh-F)**	9.64×10^{-11}	6.13×10^{-11}	1.04×10^{-10}	1.24×10^{-10}	2.34×10^{-11}
**Ag(SPh-COOH)**	2.42×10^{-11}	2.98×10^{-11}	2.77×10^{-11}	1.51×10^{-11}	2.42×10^{-11}
**Au(SPh-COOH)**	6.60×10^{-12}	5.88×10^{-12}	6.16×10^{-12}	7.77×10^{-12}	7.78×10^{-13}

The resistance measured by above methods should contain both of the intrinsic resistance and contact resistance. However, from the obtained I-V curves (Figure 4d and Supplementary Fig. 49), all samples showed Ohm contact with their electrodes. Normally, Ohm contact possesses small resistance which is neglectable compared with the intrinsic resistance of our samples (from ~kΩ to MΩ). So the contact resistance has little influence to the conductivities of OMCs.

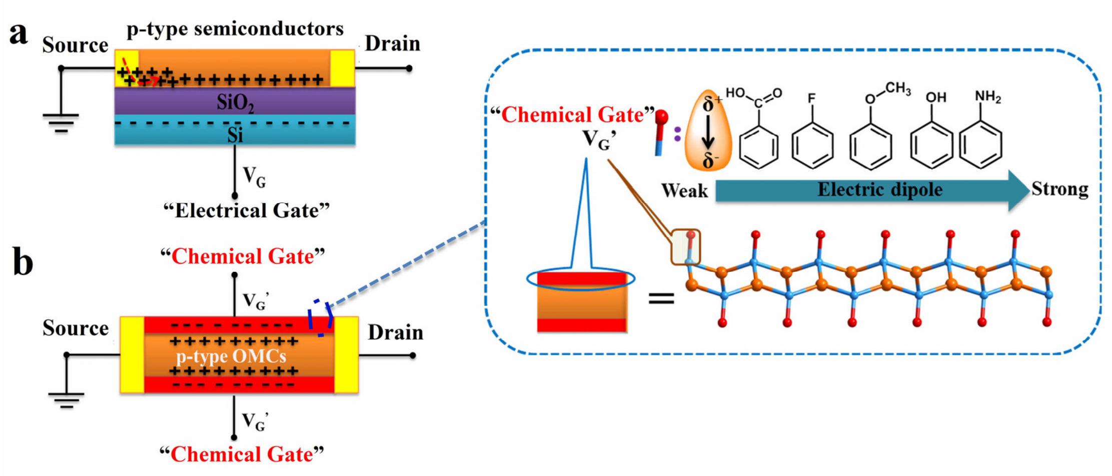

Supplementary Figure 50. A p-type FET model and the explanation of the analogous "chemical gating" properties of the electron-donating functional motifs on the benzene ring of OMCs.
